# MobsPy: A programming language for biochemical reaction networks

**DOI:** 10.1371/journal.pcbi.1013024

**Published:** 2025-05-19

**Authors:** Fabricio Cravo, Gayathri Prakash, Matthias Függer, Thomas Nowak

**Affiliations:** 1 Université Paris-Saclay, CNRS, ENS Paris-Saclay, LMF, Gif-sur-Yvette, France; 2 Université Paris-Saclay, CNRS, LISN, Gif-sur-Yvette, France; 3 Northeastern University, Boston, Massachusetts, United States of America; 4 Rice University, Houston, Texas, United States of America; 5 Institut Universitaire de France, Paris, France; National Institutes of Health, UNITED STATES OF AMERICA

## Abstract

Biochemical Reaction Networks (BCRNs) model species and their interactions via reactions. They have been extensively used in chemistry and extended to biological settings by generalizing the reactions’ kinetics. However, detailed models of biochemical processes tend to result in complex BCRN models. We present the Meta-species Oriented Biosystem Syntax (MobsPy), a language designed to simplify the modeling process using the concept of meta-species. Meta-species are constructed using a bottom-up approach from base species, which represent elementary, simple characteristics. These characteristics are then combined to create meta-species with all their complex behavior. The combined species have characteristics that are the Cartesian product of the base species’ characteristics and feature inheritance of reactions involving the base species. New reactions can involve all the states of a meta-species or only a subset that is selected via a query. In particular, reactions of meta-species can express a state change of one of the reactants. MobsPy is deployed as a Python package. We showcase its modeling capabilities by building concise models for biochemical systems from the literature.

## Author summary

The formalism of Biochemical Reaction Networks is a way to directly express a system by its species and the reactions that act upon them. Despite their natural correspondence to modeling biochemical systems, such networks may be difficult to write and maintain: complex networks have hundreds of species and reactions. We provide a new Python-based language, MobsPy, designed to simplify this process by using two concepts: First, one uses meta-reactions to specify patterns that are common to multiple reactions. Second, meta-species can be used to specify the behavior of several species at the same time. Based on these concepts the language provides a means to create species and reactions via inheritance from other species. We demonstrate the capabilities of MobsPy at the hand of several examples from the literature and compare them to existing tools.

## Introduction

A Chemical Reaction Network [1] (CRN) comprises a set of species and reactions among the species. An example is the reaction A+B→C, where *A* and *B* form a complex *C* upon interaction. While CRNs assume reaction rates to follow mass-action kinetics, i.e., proportionality to concentrations [*A*] and [*B*] in the example, the more general Biochemical Reaction Networks [2] (BCRNs) allow for more general rates as they may appear when involving complex species like bacteria.

BCRNs are a powerful formalism used for modeling biochemical systems, with the possibility of separating species interaction from reaction kinetics and immediately translating to both deterministic and stochastic dynamics. Stochastic simulations of BCRNs allow the use of algorithms like the Gillespie algorithm [3], which efficiently handles stochastic trajectories of species. BCRNs have been successful in modeling the experimental behavior of several biochemical systems, with examples being gene expression [4], phage transmission [5], controllers of gene expression [6], and metabolic engineering [7]

However, writing BCRNs can become a challenging endeavor as the complexity of the model grows [8], resulting in numerous reactions and species [8, 9]. It is not uncommon for models to reach more than 50 reactions [10, 11], with even a single genetic XOR gate reaching up to 62 reactions with 44 species [11]. As models expand, their complexity can lead to errors during the modeling process and in subsequent model updates [8]. Lopez, Muhlich, Bachman, and Sorger [8] examined existing literature and found discrepancies between descriptions of models and the actual provided models. In response to the challenges posed by increasingly intricate BCRN models, researchers have pursued the development of numerous specialized tools and languages aimed at modeling CRNs and BCRNs [8, 9, 12–20]. Not all of these modeling tools prioritize reaction syntax, with some focusing primarily on simulation. We briefly sketch some of these tools as examples: COPASI [15] is a GUI-based framework written in C++ that provides deterministic and stochastic simulations of CRNs among several analysis and parametrization methods. BasiCO [12] is a Python API that allows COPASI to be used directly in Python. Antimony [18] is a language that allows one to specify models in succinct human-readable code with language features that ease the creation of shareable modules. Related is the simulation framework Tellurium [19] that allows specifying models in Python code, including entries in Antimony, and provides extensive stochastic and deterministic simulation, analysis, and parametrization support. As a simulation backend, it uses libRoadrunner [20]. Both COPASI and Tellurium provide model export to the standardized SBML format [16] with the possibility to run simulations in different backends. iBioSim [17] is a GUI-based BCRN simulation tool that also includes features such as compartments and model generation from genetic design entries. Common to the above tools, however, is that they lack means for reaction modularity. Several tools featuring reaction modularity already exist in the literature, including rule-based languages such as Kappa [13], BioNetGen [21], PySB [8], and STOCHSIM [22]. Additionally, dedicated tools support the implementation of these languages, such as SmolDyn [23] and VCell [24], both of which integrate BioNetGen. BioCRNpyler provides abstractions in terms of mechanisms, components, and mixtures.

Here we introduce MobsPy (Meta-Species Oriented Biosystem Syntax in Python), which is a new Python-based programming language aimed at decreasing the complexity of BCRN model entry, simulation, and analysis.

For model entry and analysis, MobsPy uses the concepts of meta-species and meta-reactions. A meta-species is a set of species, with a species representing the state of the meta-species, i.e., a particular assignment of characteristics and meta-reactions are reactions between meta-species. Meta-species can be directly defined or constructed via the Cartesian product of predecessor meta-species. By forming the product, an orthogonal state space is created, with each predecessor species representing a base in this state space. Moreover, meta-species inherit all reactions from their predecessors. It allows for an approach where one defines basic meta-species with reactions that act upon them and, in a second step, assembles them into a product meta-species with all combined reactions.

Unlike rule-based languages, MobsPy’s object-oriented approach allows state queries that reference not only the species containing a given state but also all its inheritors simultaneously. Additionally, meta-species states can be queried dynamically in resulting data without the need to define observables. Moreover, MobsPy simplifies modeling reproduction-like reactions (e.g., R→2R, 2R→3R ) by automatically assigning states to unmatched meta-species based on the order of reactant-product transformations. In contrast, for this reaction type, rule-based languages require explicitly defining individual reactions for each state.

In contrast to BioCRNpyler’s species, all reactions in MobsPy can be directly inferred from the model’s code. Moreover, akin to the provided BioCRNpyler classes, meta-reactions in MobsPy can be encapsulated within Python functions, enabling sharing and reuse throughout different models.

MobsPy focuses on syntax simplification and readability, similarly to Antimony which focuses on a more readable alternative to SBML. However, Antimony does not provide reaction modularity and does not attempt to streamline the number of reactions in a model. Similarly to Antimony, MobsPy has mid-simulation model changes implemented.

MobsPy further incorporates the Pint Python module [25] to allow for units in rates, counts, and concentrations that are checked for consistency and appropriately converted for simulation. An example is Michaelis–Menten kinetics, where BioCRNpyler verifies units of both the Michaelis constant and the limiting rate. Additionally, it provides a means of specifying non-mass-action rates directly via arithmetic expressions in Python and automatically checks if the resulting unit is consistent.

For simulation, MobsPy internally generates SBML files and runs deterministic (ODE) or stochastic (Gillespie and Gibson–Bruck) simulations via the COPASI bindings from BasiCO. Simulation via other backends, such as libRoadrunner, is possible via SBML export. Additionally, the provided features available in the MobsPy Python module are: (i) Parametric sweeps and the utilization of multiple CPU cores when appropriate. Parameters, as well as meta-species, are automatically assigned names that are derived from their corresponding Python variables unless otherwise specified. It eliminates the burden of using strings to name them redundantly. Further, conducting a parametric sweep is effortlessly achieved by assigning multiple values to a parameter. (ii) Event handling, where MobsPy accommodates the addition of time-based or condition-based events that dynamically modify meta-species counts or parameter values during simulations. In particular, condition-based events offer the flexibility to end simulations under specific conditions. (iii) The possibility of enchaining simulations is expressed in MobsPy as a simple summation of simulation objects. Furthermore, each simulation within the concatenated one can be simulated either deterministically or stochastically. A particular use-case of this is simulation via the computationally more expensive stochastic solver until a specific condition is reached and then switch to a faster ODE solver.

## Design and implementation

### The MobsPy language

**Fig 1:**
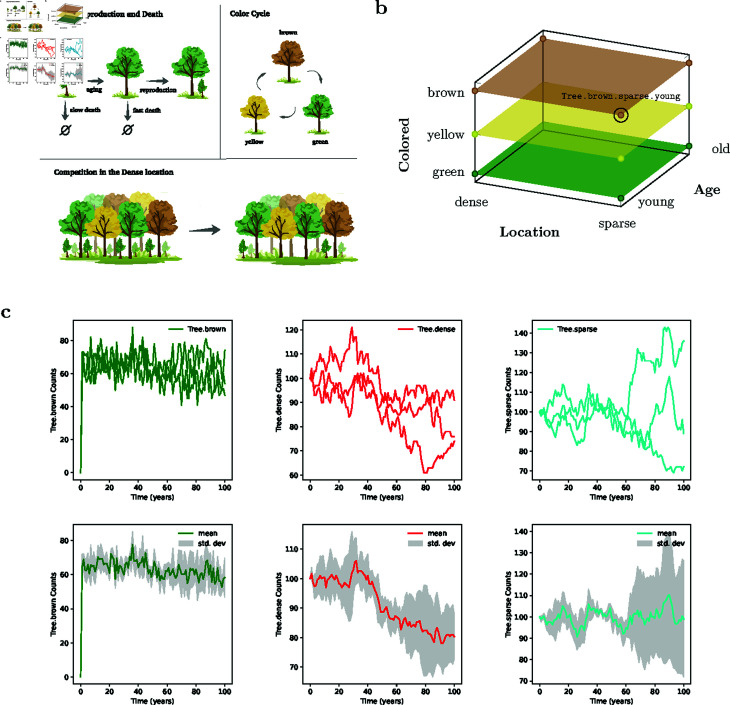
(a) System dynamics: schematic representation of all the reactions in the Tree model, which are aging, reproduction, color cycle, and competition. **(b)** Meta-species  Tree created by multiplication of the three base-species  Age,  Location, and  Colored. One of the twelve species generated,  Tree.brown.sparse.young, has been labeled. **(c)** MobsPy default plots after simulating the Tree model (*n* = 3 stochastic runs). In the top row of the panel, individual runs are shown. The bottom plots depict the mean and standard deviation.

We discuss the main features of MobsPy along with a running toy example. Consider a system of two locations where trees grow (Fig 1a): one location where trees grow and reproduce within a dense population and one where they thrive in a sparser environment. Trees die and age, with young trees in the dense environment dying at a higher rate due to competition for resources and space. Further, a tree’s leaf colors cyclically change from green to yellow to brown and back to green.

MobsPy simplifies the definition of BCRNs through a bottom-up approach. Base meta-species work as foundational building blocks that encapsulate elementary functions. The base meta-species are then combined to form more complex species, inheriting both reactions and states from their foundational predecessors. We start with modeling the aging process. A base meta-species named Age is introduced, and the reaction (>>) operator is applied to add the transition from a young Age to an old Age with a given reaction rate that is per-default mass-action and has a rate constant specified within subsequent brackets. The construct is now ready to be used for the specification of more complex meta-species via inheritance.



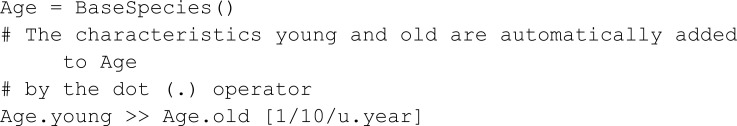



Reversible reactions are expressed by applying the Rev[] operator to a reaction, followed by a forward and backward rate in brackets. New characteristics of a meta-species are introduced via the dot (.) operator. It can be done inside (like in the Age example) or outside of reactions. We will use the latter in our running example by adding characteristics to a Colored and a Location meta-species.

Colored, Location = BaseSpecies()

Colored.green, Colored.yellow, Colored.brown

Location.dense, Location.sparse

It is further possible to create meta-species and assign reactions to them without introducing new characteristics. As an example, consider a Mortal meta-species to model a death reaction. In the reaction, we make use of Zero, a special meta-species that represents the absence of reactants or products in a reaction.

Furthermore, MobsPy allows one to specify reaction rates that depend on the characteristics of the reactants by replacing the reaction rate constant with a function whose arguments are the reactants. In case the returned expression contains a reactant (e.g., in lambda r1, r2: r1/(r1 + r2)) it is taken as a reaction rate. Otherwise, as in the example below, mass-action kinetics is assumed and the returned value is taken as a reaction rate constant.



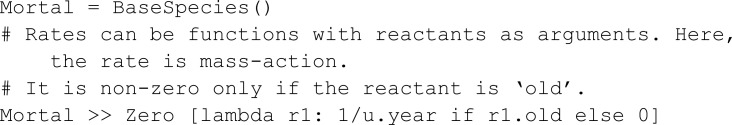



We are now ready to construct the Tree meta-species from the basic meta-species by combining the Colored, Age, Mortal, and Location meta-species through the multiplication (*) operator. The combination encapsulates the representation of leaf color, lifespan, mortality potential, and location (dense or sparse environment).

Tree = Age * Colored * Mortal * Location

The species within Tree are obtained via the Cartesian product over the sets of states of its predecessor meta-species. All characteristics of a predecessor species are accessible by a meta-species that inherits from this predecessor species. Illustrative is the view of a meta-species as a point in a multi-dimensional space (Fig 1b). A species in Tree has Age, Colored, and Location components with either ‘young’ and ‘old’ in the Age component, ‘brown,’ ‘green,’ or ‘yellow’ in the Colored component, and ‘dense’ or ‘sparse’ in the Location component. In our example, 12 species of the form Tree.(young|old).(brown|green|yellow).(sparse|dense) are subsumed within the Tree meta-species.

The dot operator has different semantics in reactants and products. When used in a meta-species that is a reactant, it acts as a query and refines the meta-species to those species that possess this characteristic. For example, the reactant Tree.young is the set of species of the form Tree.young.(brown|green|yellow).(sparse|dense). Conversely, when used in products, the dot is used to specify a change of characteristics. For example, the product Tree.young is the respective reactant with characteristics age set to ‘young’. Following these rules, in the running example, the meta-reaction Age.young >> Age.old is compiled into the following reactions:



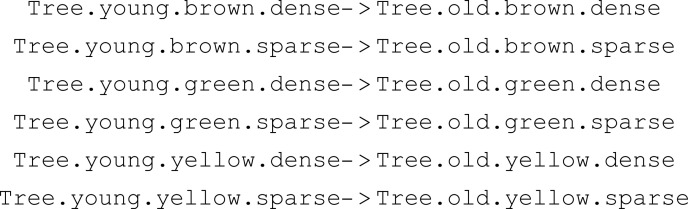



**Fig 2:**
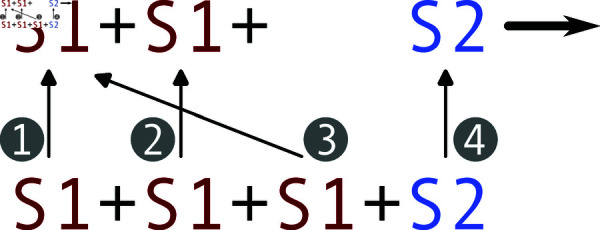
Matching products with reactants for the example meta-reaction 2 * S1 + S2 >> 3 * S1 + S2 where S1 and S2 are distinct meta-species. MobsPy starts with the leftmost product and matches the leftmost reactant with the same meta-species (indicated by an arrow) in step 1. The process is repeated with the next reactant (step 2). For the next product (step 3), MobsPy cycles back to the first reactant. Finally both S1 are matched in step 4.

Since the dot operator on products only defines characteristics of the reactant that are to be changed, it is essential to specify which product originates (and thus inherits default characteristics) from which reactant. While a matching of products with reactants is unambiguous for reaction S1.a >> S1.b with meta-species S1, matching in reactions with multiple products and reactants is *a priori* ambiguous. Starting with the first product, MobsPy cycles through the reactants until it finds one that has the same meta-species as the product upon which the product is matched with the reactant. It is continued with every product until all are matched with a reactant (Fig 2).

We make use of the cyclic matching in the subsequent two reactions of the running example.



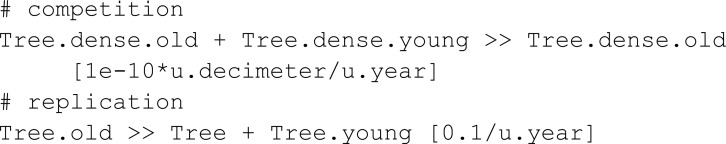



The first reaction models the behavior of old trees suppressing the growth of young trees within the dense location. The matching implies that the old Tree survives, as opposed to the young tree surviving and becoming old. The second reaction models the replication of old trees. Here, the old tree remains unchanged and creates a new tree that is identical to the old one, except being young.

The next reaction is a low-rate sexual reproduction reaction where two trees generate a new young one containing the characteristics of a parent. In this reaction, trees in the same location and color have a much higher rate for reproduction. This is archived by extracting the location of a reactant with the call operator using the name of a predecessor meta-species.



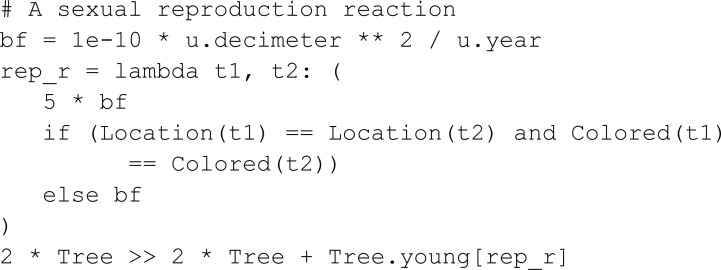



This meta-reaction adds 144 reactions to the model and shows how MobsPy’s automatic round-robin order allocation can simplify the definition of reproduction-based reactions.

It remains to specify the cyclic color transition of the Tree species over time. This is achieved via the characteristics (c) function of a meta-species: S.c(s) is equal to S.v, where v is the value of the string variable s. We use this method to loop over the colors.

colors = [’green’, ’yellow’, ’brown’]

for color, next_color in zip(colors, colors[1:] + colors[:1]):

Tree.c(color) >> Tree.c(next_color) [10/u.year]

For initial conditions, the call ( () ) operator is used to set initial counts of meta-species. In case the meta-species contains multiple species with different characteristics, the assignment is made to the default characteristics of the meta-species, which is the one that was added first. For example, the default characteristics of Colored is green, and the default characteristics of Age is young. Example initializations for the tree model are:



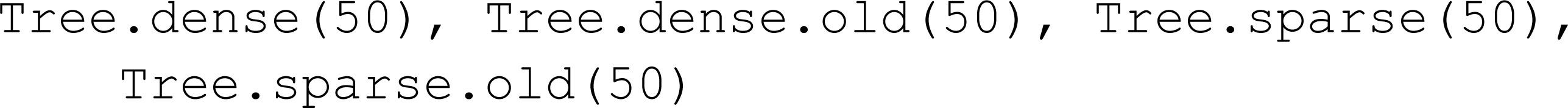



MobsPy distinguishes between meta-species solely used to construct other meta-species and those that will be used in a simulation. For example, Age, Colored, and Mortal are only used to construct Tree, while the Tree meta-species contains the species whose behavior is to be simulated. In MobsPy, species that are to be simulated are passed to the Simulation constructor separated by |. The constructor then compiles these meta-species and the meta-reactions that act upon them into sets of species and reactions. For the running example, this is achieved via:

MySim = Simulation(Tree)

Finally, stochastic (Gillespie) or deterministic (ODE) simulations are run for a given duration, with units of the model being checked for consistency and default plots being displayed (Fig 1c).



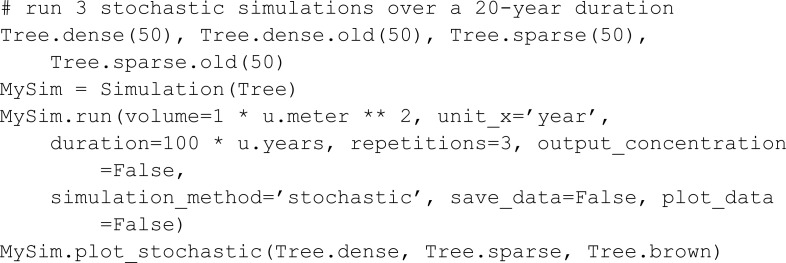



## Results

### Models

Next, we present MobsPy models from several BCRNs in the literature.

#### Mutual annihilation protocol.

In some of the authors’ previous work [26], we proposed a stochastic bacterial system for reconstructing and amplifying a chemical signal constituted of two species A and B. If A is in excess, the signal is logical 1, while an excess of B encodes a logical 0. Desired is a large gap between concentrations of A and B by a mutual annihilation reaction between A and B. Concurrently, the concentrations of both species are amplified by replication reactions.

We show a simulation of the system in two phases: one deterministic growth phase that establishes high concentrations of species A and B with the replication reactions and without the annihilation reaction, and a subsequent stochastic phase that enables the annihilation reaction. The first phase can be defined as follows:

from mobspy import

* import os

A, B = BaseSpecies()

# Replication reactions

A >> 2 * A [1.05 / u.h]

B >> 2 * B [1 / u.h]

# Initial counts

A(1 / u.ml), B(1 / u.ml)

# First phase

S1 = Simulation(A | B)

S1.duration = 3*u.h

S1.volume = 1*u.ml

The second phase is modeled by adding the annihilation reaction to meta-species A and B and creating a new simulation object S2 with the additional reaction. The complete two-phase model S is obtained as the sum of S1 and S2.

# Annihilation reaction

A + B >> Zero [0.1/u.h]

S2 = Simulation(A | B)

S2.duration = (A <= 0) | (B <= 0)

S2.method = ’stochastic’

S2.volume = 1*u.ml



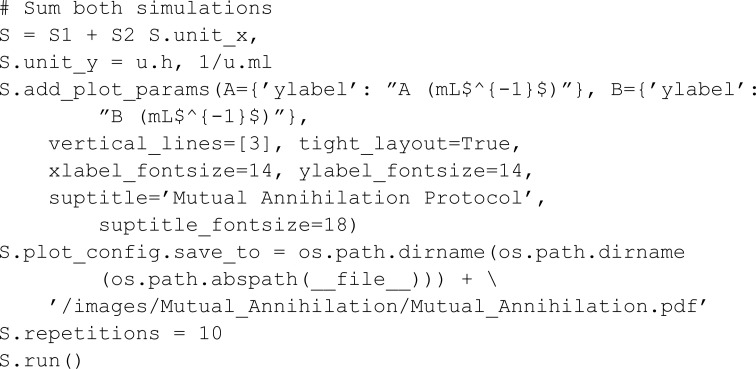



**Fig 3:**
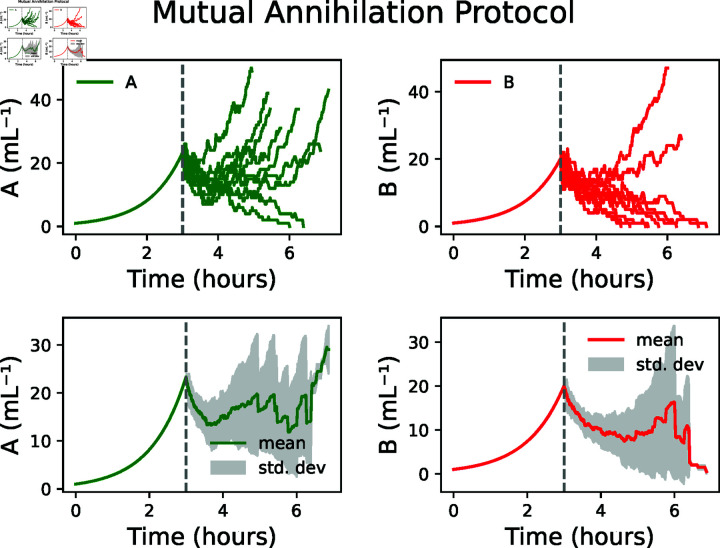
Simulated dynamics of the Mutual Annihilation protocol [26]. By default, for stochastic simulations, MobsPy plots the runs of each species as well as the mean and standard deviation. The dotted gray line represents the transition from the deterministic only-growth phase to the stochastic annihilation. The species A survives in the majority of the runs.

The results (Fig 3) are consistent with what is expected from our previous analysis: Since the second phase is stochastic, there is a non-zero, although small, probability that the concentration of B surpasses that of A and B survives even though it is in a lower concentration than A at the beginning of the second phase.

#### Bistable gut inflammation detector.

Riglar *et al.* [27] implemented a system designed to detect intestinal inflammation within the mammalian gut. It is composed of a trigger element and a memory element. The trigger element detects the presence of one of the gut inflammation byproducts (tetrathionate, TTR). The introduction of TTR results in the phosphorylation of TtrS which then triggers a chain reaction that up-regulates the production of Cro. The memory element is based on the interaction of two proteins, Cro and CI, which function as mutual repressors of each other, establishing a state of bistability where either Cro is high and CI low, or vice versa. The chemical species Cro then represses CI, going from a low-Cro high-CI state to a high-Cro low-CI state. After the state transition, the high-Cro low-CI state remains stable even if the inflammation byproduct TTR leaves the system. It returns to a low-Cro high-CI state if CI is added to the system. In MobsPy we define:



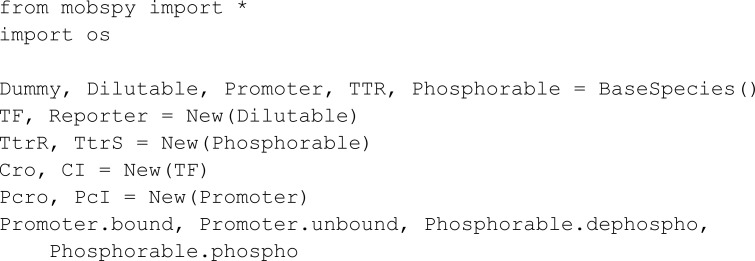



The phosphorylation reactions that trigger the production of cro are specified via:



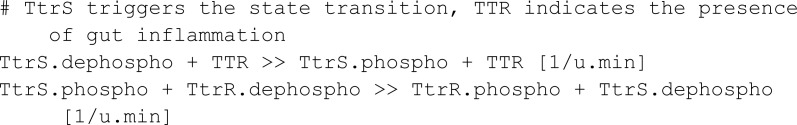



TtrR.phospho >> TtrR.dephospho [5*1e-3*1/u.second]

TtrR.phospho >> Cro + TtrR.phospho [50*0.02*1/u.min]

To simulate the delayed addition of CI, we use a placeholder meta-species Dummy. Starting with an initial count of zero, the Dummy meta-species is set to one during the execution of the simulation, triggering an influx of CI.



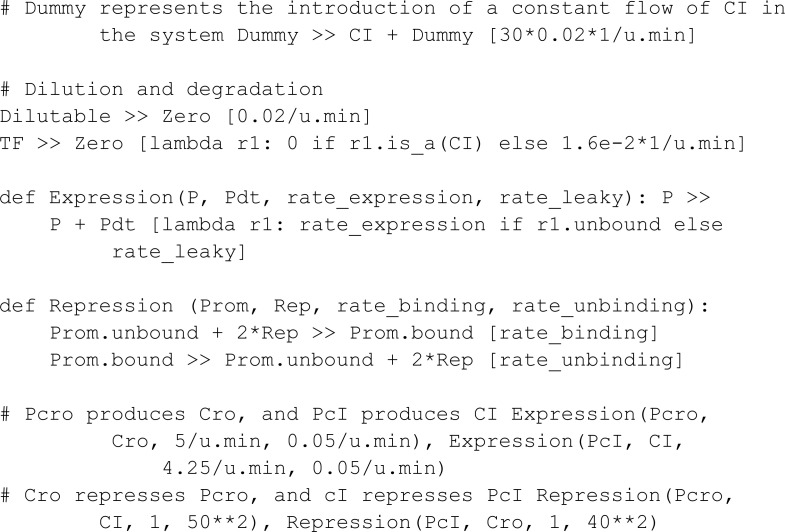



One can validate that the introduction of the inflammation byproduct results in the low-Cro state transitioning to a high-Cro state that remains after the event that removes the byproduct. MobsPy’s event syntax uses the keyword with and the Simulation object with the methods event_time for time-based triggers or event_condition for conditional-based triggers. The species concentrations to be set in an event are stated inside the with block, similar to setting initial values.



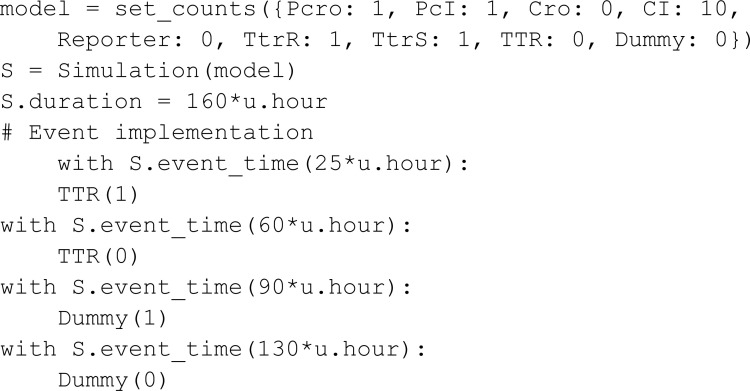





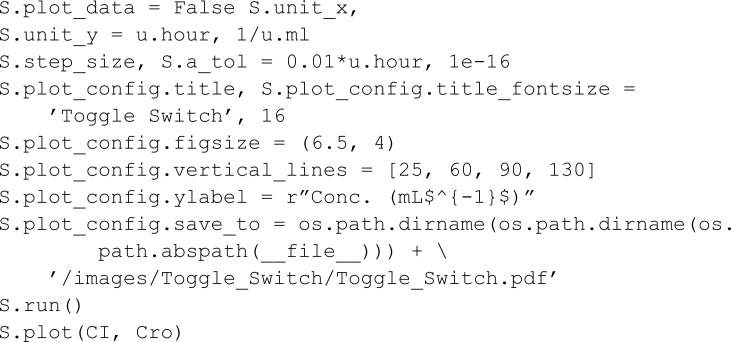



**Fig 4:**
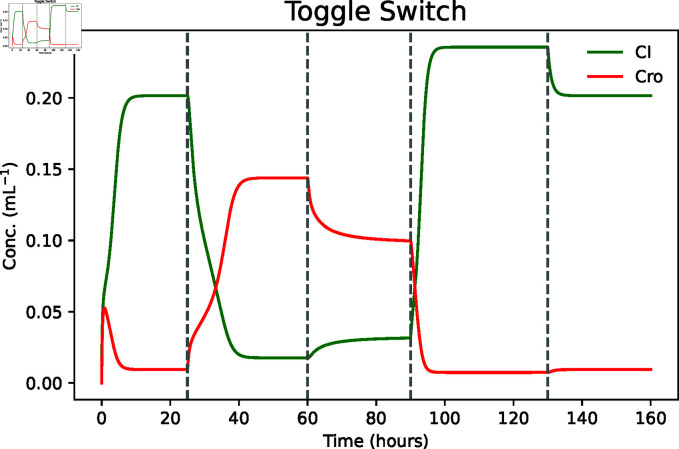
Results obtained from the Bistable Gut Inflammation Detector model. The exposure of the system to TTR (at 25 hours) shifts the system to a high-Cro state, which remains even after the removal of TTR (at 60 hours). The system only returns to a low-Cro state when introducing CI (at 80 hours).

The simulation (Fig 4) shows that the introduction of the inflammation byproduct (at 25 hours) results in a transition from a low-Cro high-CI state to a high-Cro low-CI state, with the latter state persisting after the inflammation marker removal (at 60 hours). Moreover, the reintroduction of CI results in a transition back to the low-Cro high-CI state (at 80 hours).

#### Phage transmission system.

Pathania *et al.* [5] studied a bacterial system that utilizes bacteriophages for transmitting antibiotic resistance. In their work, a bacterial population (Donor) secretes phages carrying an antibiotic resistance gene, which infects a bacterial population (Receiver) devoid of such.

Cell duplication was modeled using two distinct resources (R1 and R2). Resources R1 and R2 are consumed for bacterial replication with a faster uptake rate for R1. Further, R1 is also the only resource consumed by a Donor for phage production. The discrepancy in consumption rates leads to the depletion of R1 with only R2 being available afterward.

Like the Mutual Annihilation protocol, this experiment is sectioned into two phases with antibiotics being introduced in the second phase. If Donor species can produce sufficient phages to transmit the antibiotic resistance to the Receiver species, the Receiver species will survive the introduction of antibiotics; otherwise, they die.



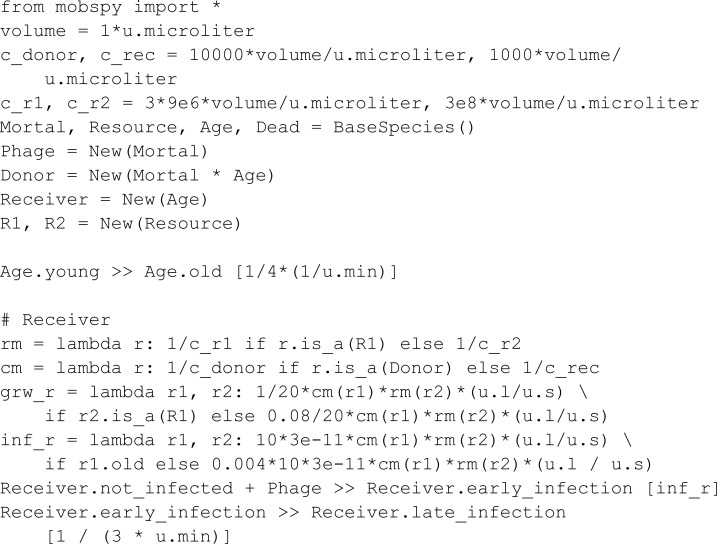



In the model, a Phage can bind a dead Receiver, resulting in the loss of a phage. We keep track of dead Receiver species through a reaction transforming a Receiver into Dead.



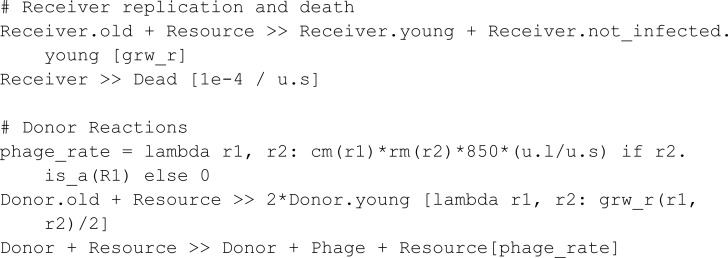





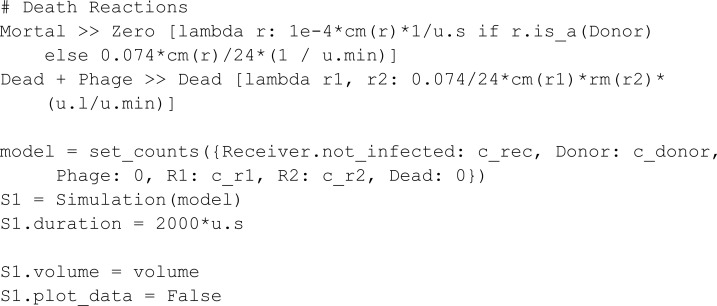



In the second phase Antibiotics is introduced into the system:



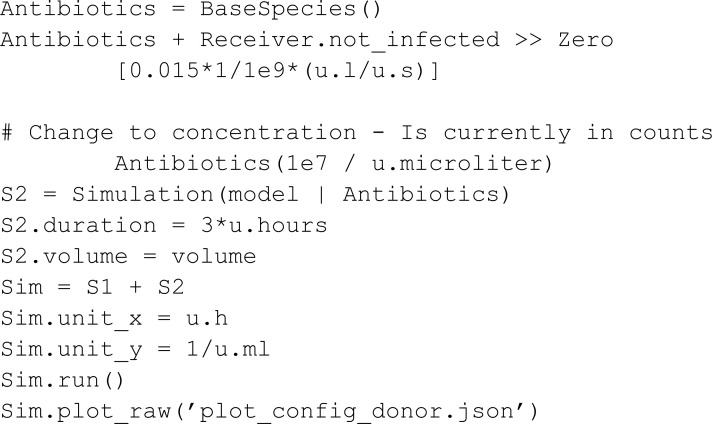



**Fig 5:**
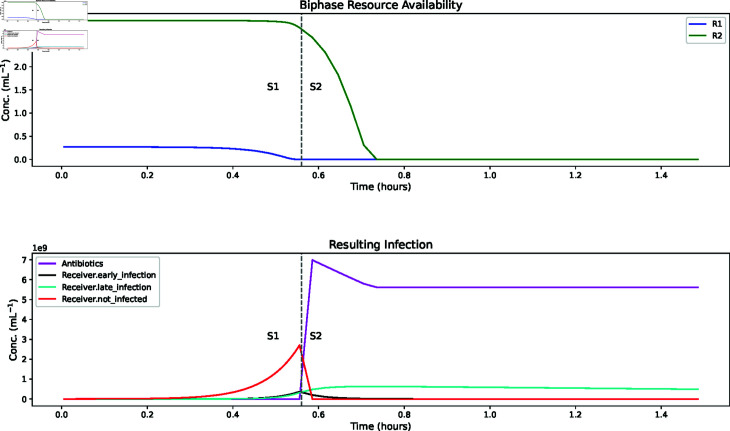
Simulation results for the phage communication circuit from Pathania *et al*. [5]. The gray line separates between the simulation phase dedicated to resource dynamics (S1) and the simulation phase with an antibiotic introduction (S2) **Top** The resource availability achieved in this model. Resource R1 is consumed faster than R2, resulting in a window of time where mostly R2 is available, and R1 is depleted. **Bottom** Resulting infection progress. Only infected Receiver species remain.

The simulation results (Fig 5) are in accordance with the original work: One observes that the Receiver population has survived the introduction of Antibiotics due to a successful transmission of antibiotic resistance.

#### Synchronized cycles of bacterial lysis.

Omar Din *et al.* [28] designed a bacterial system for *in vivo* drug delivery through the synchronized death of a bacterial population. Each bacterium produces *N*-acyl homoserine lactone (AHL) and secretes it into the medium. Consequently, the concentration of AHL increases as the number of bacteria increases. If the concentration of AHL passes a threshold, it triggers a lysis process via quorum sensing that kills most of the bacterial population, leaving only a slim fraction compared to its pre-lysis size. This process creates a cyclic behavior.

Model-wise, meta-species LuxI is a protein that dictates the secretion rate of AHL by a cell. Meta-species Lysis models the intracellular enzyme responsible for the lysis process. This enzyme inhibits peptidoglycan biosynthesis [29], causing cellular death. The production of Lysis increases as a consequence of an increase in the AHL concentration. Bacterial cells are modeled by the meta-species Cell.



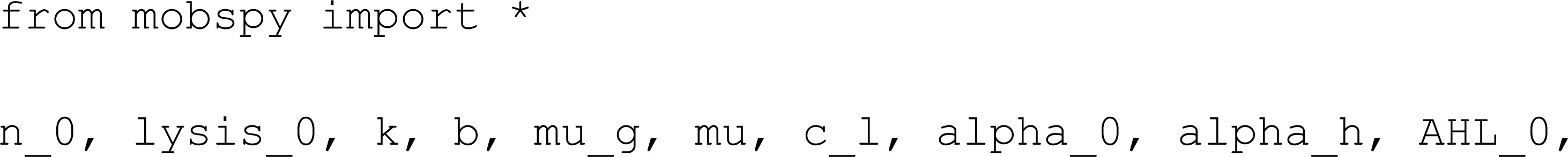





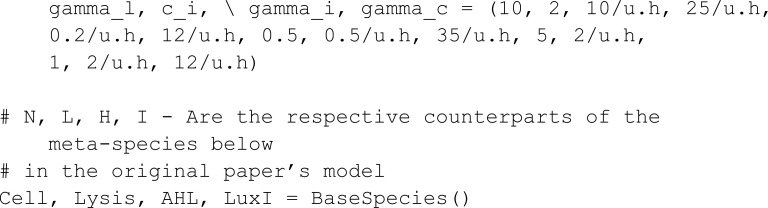



The BCRN does not exclusively use mass-action-kinetic reaction rates. In MobsPy this can be expressed by Python functions that return expressions that include reactants (see the following example). Such expressions are compatible with units and checked for consistency.



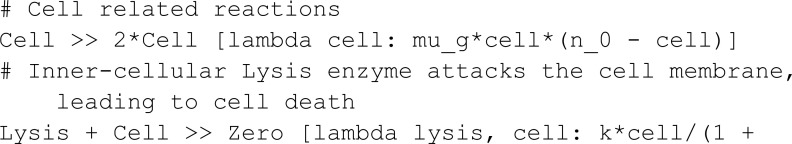





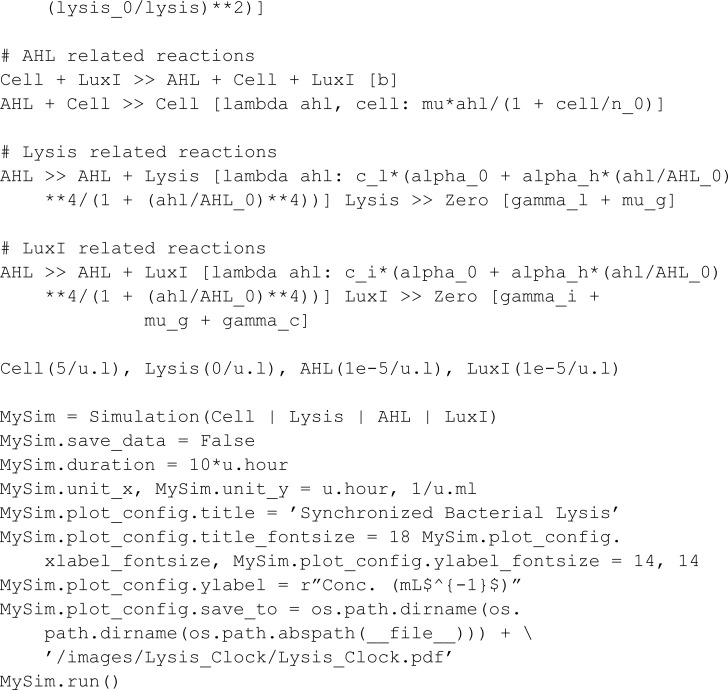



**Fig 6:**
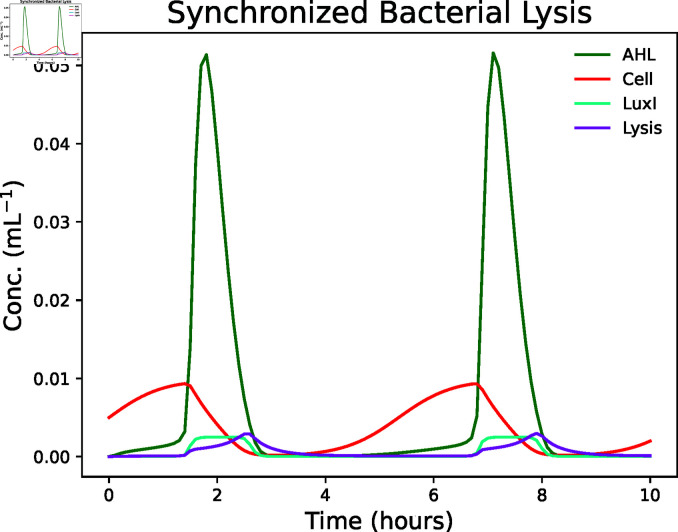
Simulation results of the quorum sensing circuit by Omar Din *et al*. [28]. The simulation shows a synchronized lysis of bacteria. As the AHL levels and population size increase, eventually, a threshold is reached, and the lysis process starts killing the majority of the population. The cycle then repeats.

Omar Din *et al.* [28] experimentally showed that the number of cells oscillate over time, which is reproduced by the above model (Fig 6).

#### Logical XOR gate.

**Fig 7:**
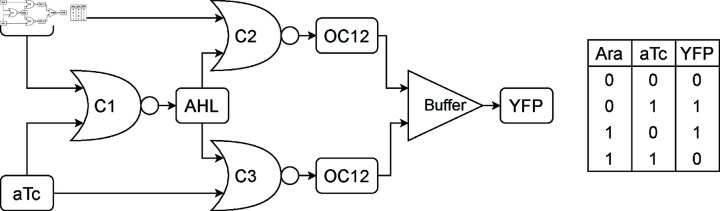
Left schematic of the XOR gate implementation [11]. **Right** Truth table of the circuit, equivalent to the specification of a XOR gate.

Tamsir *et al.* [11] implemented an XOR gate across three bacterial colonies, each colony functioning as a NOR gate (Fig 7). In the BCRN model, the XOR and NOR gates take the concentration of two species as inputs and produce another species as output. NOR gate C1 takes inputs Ara and aTc, outputting AHL. NOR gates C2 and C3 both take AHL as input, with C2’s second input being Ara and C3’s second input being aTc. The outputs of C2 and C3, named OC12, serve as input for a buffer that outputs yellow fluorescent protein YFP.

We define a meta-species Location, with characteristics c1, c2, c3, and c4 representing the location of the three colonies and the buffer. The NOR gates are fixed in place; in contrast, the BCRN species Ara, aTc, AHL, and OC12 can move between colonies, serving as inputs to subsequent gates. We model this with a species Movable that inherits from Location and that contains location transitions.

from mobspy import *

import numpy as np

max_plas, max_pbad, max_ptet = (1, 1, 1)

Mortal, Location = BaseSpecies()

Location.c1, Location.c2, Location.c3, Location.c4

PBad, PTet, Pcl, PLas, Movable = New(Location)

Ara, aTc, Cl, YFP, AHL, OC12 = New(Mortal*Movable)



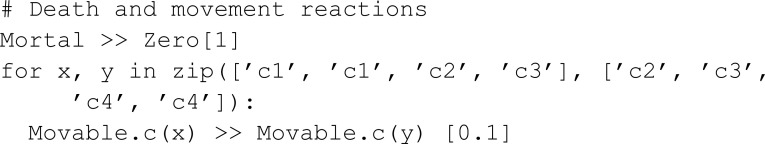



In this model, we measure the YFP output concentration for different input concentrations through a parametric sweep using the ModelParameters constructor. If a parameter has multiple values, MobsPy will run a parametric sweep using all values. Moreover, if multiple parameters have multiple values, MobsPy sweeps all possible combinations. In the model below, ara_r and atc_r are examples, controlling the concentration of Ara and aTc respectively.



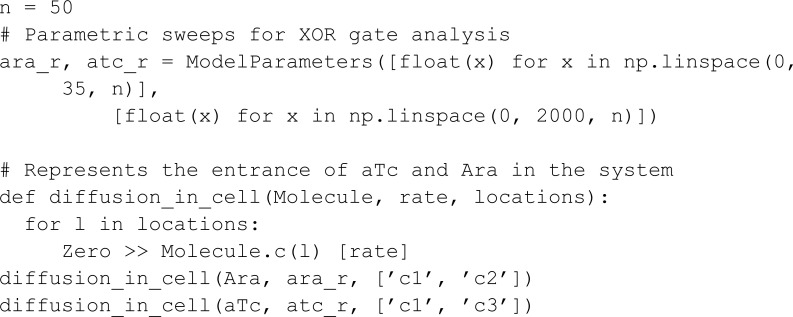



The function promoter_activation represents the production of a BCRN species. It takes arguments such as the Promoter (the chemical species binding to NOR gate inputs for production), the Ligand (a NOR gate input), constants regulating input binding tf_max, n, and K_d, an expression controlling production rate protein_production_rate, and a list of locations where this reaction occurs locations. The third argument (Protein) is the species produced.



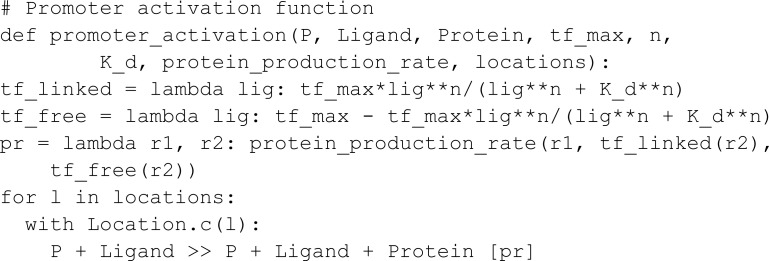



The advantage of promoter_activation is that Ara, aTc, and AHL undergo similar reactions within their respective bacterial colonies. However, these reactions involve different rate expressions, constants, and locations. MobsPy retains similar aspects within the function definition, and the differences can be passed as function arguments.

The function inverter_wire represents the NOT part of the NOR gate. The first argument (P) is a meta-species that binds to the meta-species in the second argument (R) to repress the production of the meta-species in the third argument (Signal). When the concentration of R is high, Signal will not be produced, and when the concentration is low, Signal will be produced.



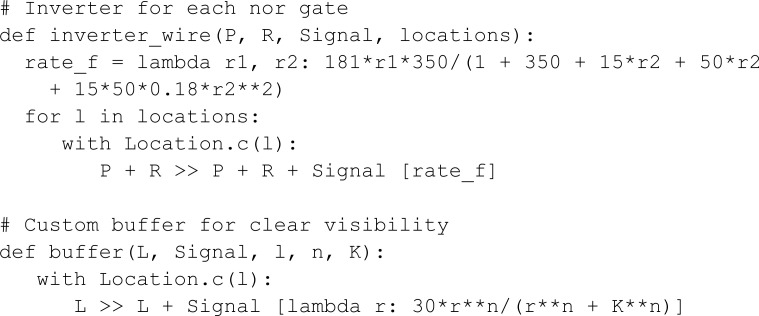



We finish the model by passing the arguments to the respective functions:



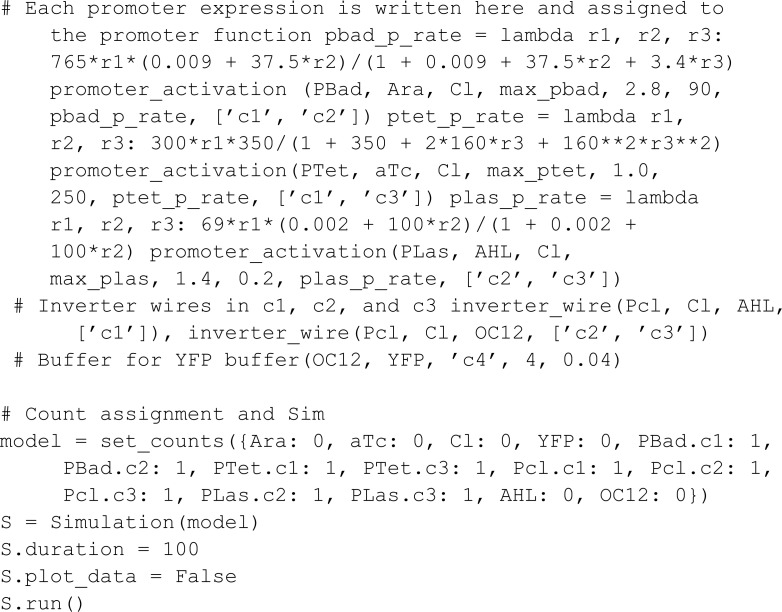



**Fig 8:**
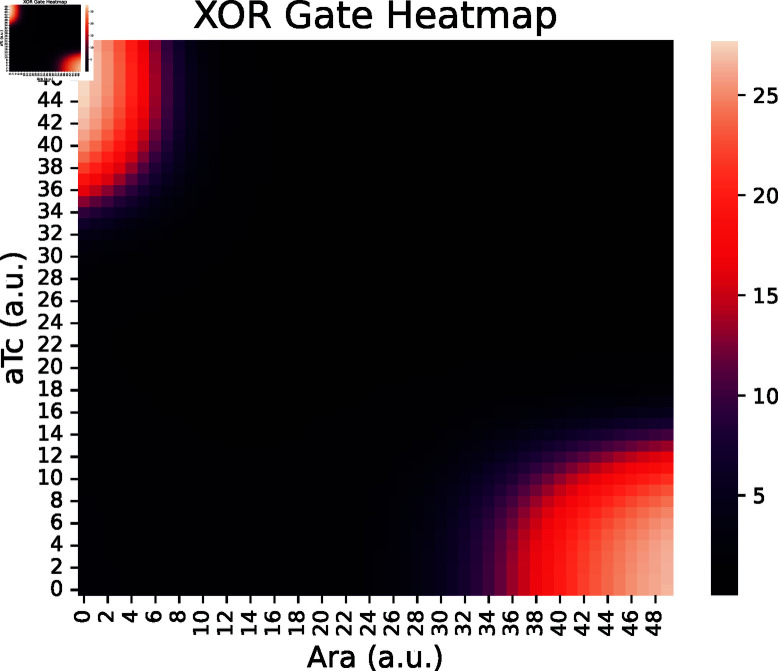
Heatmap showing the simulated output of the XOR circuit by Tamsir *et al*. [11]. Only the regions with low-aTc and high-Ara as well as high-aTc and low-Ara show a high output as expected from a XOR gate.

The simulated data (Fig 8) clearly shows the characteristics of a XOR gate.

### Comparison with related simulation frameworks

**Reaction modularity.**

MobsPy targets the concept of reaction modularity, i.e., a means to represent sets of reactions rather than individual reactions. The inspiration for this concept is drawn from the programming principle of modularity and object-oriented programming [30], where developers construct reusable code modules and classes with the goal of faster and less error-prone software development. By adopting a modular approach, both in programming and in the realm of BCRN modeling, the redundancy of re-implementing code or reactions is mitigated. This practice offers substantial benefits, including facilitating updates, where modifications are required only in one module, and enhanced debugging through a reduction in the number of lines of code or reactions that necessitate inspection.

Achieving reaction modularity within BCRN models is pursued through a number of languages such as Kappa [13, 31], BioNetGen [14], and PySB [8]. In these languages, modularity is provided through a rule-based system that emphasizes the interactions between binding sites or domains within chemical species. Notably, chemical species sharing similar domains or binding sites are governed by the same rules, thus enabling the definition of multiple reactions through these shared rules. Thus, updating several reactions becomes as simple as changing a rule.

MobsPy focuses on an object-oriented approach where states and reactions are shared through inheritance. Although multi-state molecules are already used in STOCHSIM [22], BioNetGen [21], and BIOCHAM [32], they do not have the benefit of inheritance. The inheritance allows for multi-species queries of all species that share inheritors simultaneously.

BioCRNpyler [9] takes a different approach than modularity via binding sites. It uses mechanisms, components, and mixtures to achieve reaction modularity. Components can encompass several species, and each species can be of multiple components. Mechanisms define the reaction schema that applies to components, while mixtures establish the modeling context by constructing CRNs using components and mechanisms. BioCRNpyler’s models rely on an extensive library of predefined Python classes. Notably, the components each mechanism interacts with are not explicitly presented in the model’s code, requiring users to look into the assignment of reactions from the Python classes or to decipher proposed models from code. Moreover, extensions beyond the default classes are challenging, as the user must implement their modules using object-oriented programming. The method of rate definitions provided in an extension is also constrained to mass-action, Hill expressions, and similar options, with no provision for writing custom rate expressions [10].

For the simulation perspective of tools with reaction modularity, it is worth noting that out of the previously cited tools with reaction modularity, Kappa and BioNetGen are capable of simulating models created within their frameworks. On the other hand, BioCRNpyler and PySB, generate SBML files that can be integrated with dedicated simulation tools.

**Physical units.**

Like reaction modularity, the use of physical units helps reduce errors in complex models. It is particularly the case within the domain of synthetic biology, where unit systems play a central role [33] due to the interplay of many components from potentially different labs. Indeed, the iGEM registry, a registry used for genetic data in synthetic biology, presents all their rates with units when applicable [34]. Current tools that provide reaction modularity, like Kappa, BioNetGen, and BioCRNPyler [10, 31, 35], rely on the modeler to ensure unit consistency. MobsPy assists with unit consistency by automatically converting rates, concentrations, and counts to a standardized unit system. Additionally, when multiple parameters with units are used in a rate expression, MobsPy verifies unit consistency by performing automatic unit operations to ensure the resulting expression has coherent units.

In the rest of this section, we compare MobsPy with Kappa and BioCRNpyler in depth at the hand of examples from their tutorials rewritten in MobsPy.

#### Comparison to Kappa.

We take the first model in the Kappa tutorial [31] (we removed the definition of observations for brevity). The model involves phosphorylation of the binding sites of a BCRN species C. The phosphorylation occurs twice, firstly when a species C reacts with A bound to a species B, and again when C reacts with A alone:



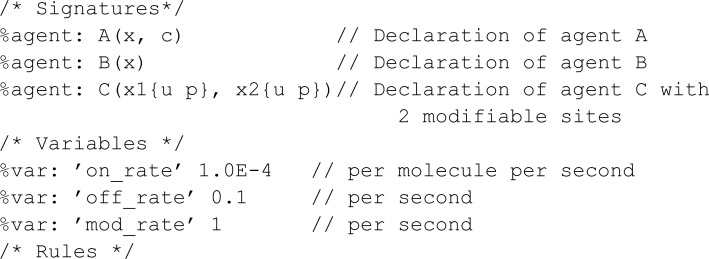





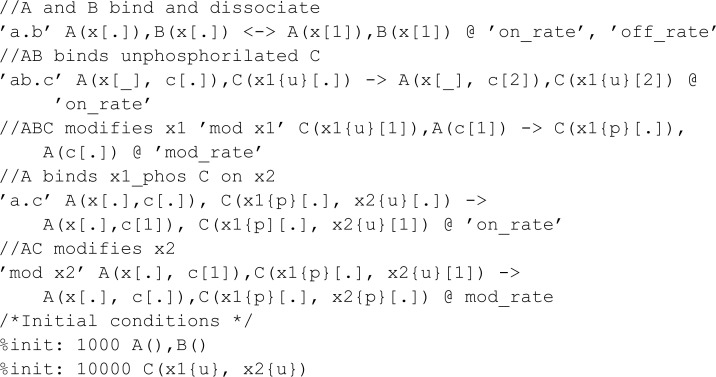



As Kappa revolves around the occupancy of binding sites of a BCRN species, we represent the binding sites through meta-species Link1 and Link2. The meta-species Link1 is reserved for interactions involving A and B, while Link2 is reserved for interactions involving A and C. Both Link1 and Link2 meta-species have two characteristics, not linked (nl_2 and nl_1) and linked (l_2 and l_1). Further, the meta-species Phos represents the phosphorylation states.



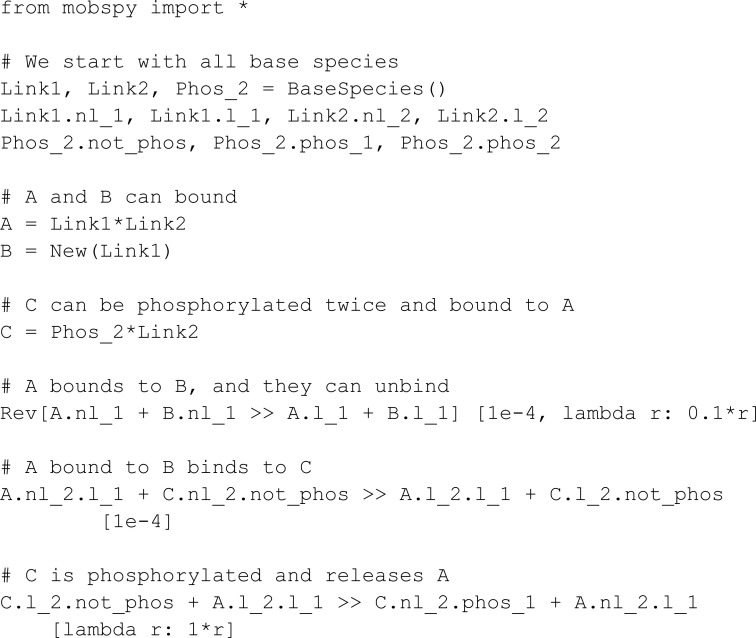





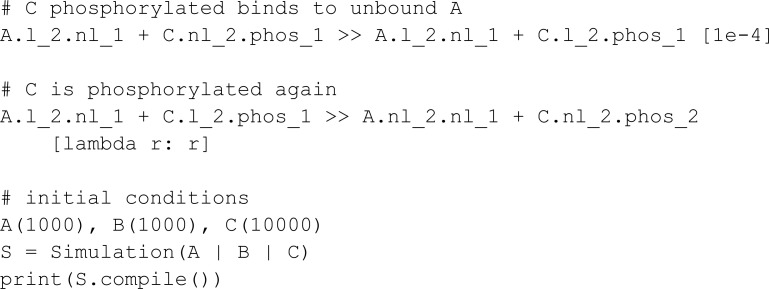



Furthermore, the Kappa graph-based algorithm also alleviates the burden of state space explosion by using graph connections instead of states for representation, while MobsPy simply focuses on syntax simplification. For instance, when representing the links of polymers in reactions, MobsPy would still be capable of writing the model. However, compilation would require a larger amount of time.

#### Comparison to BioCRNpyler.

We next implement the two initial models from BioCRNpyler GitHub repository [10]. The first basic example is:



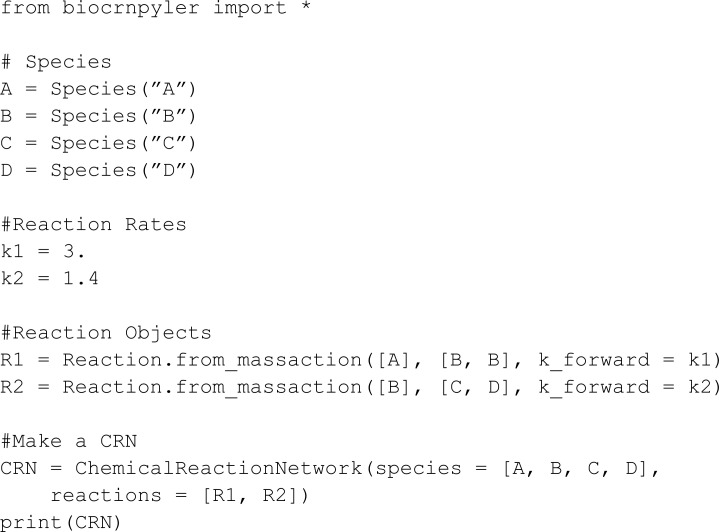



The initial model consists of a simple BCRN where a BCRN species A reacts alone, resulting in two of species B, and B reacts alone, resulting in species C and D.

from mobspy import *

A, B, C, D = BaseSpecies()

A >> 2*B [3]

B >> C + D [1.4]

S = Simulation(A | B | C | D)

print(S.compile())

Unlike the model provided in BioCRNpyler’s GitHub [10], MobsPy’s automated variable naming eliminates the step of naming a BCRN using a string equal to its variable name. Additionally, in BioCRNpyler, to define reactions with mass action kinetics rates, the user must call the method from_massaction from the class Reaction. MobsPy’s expressions and default mass-action kinetics make this step more succinct.

The second example showcases a more complex network:



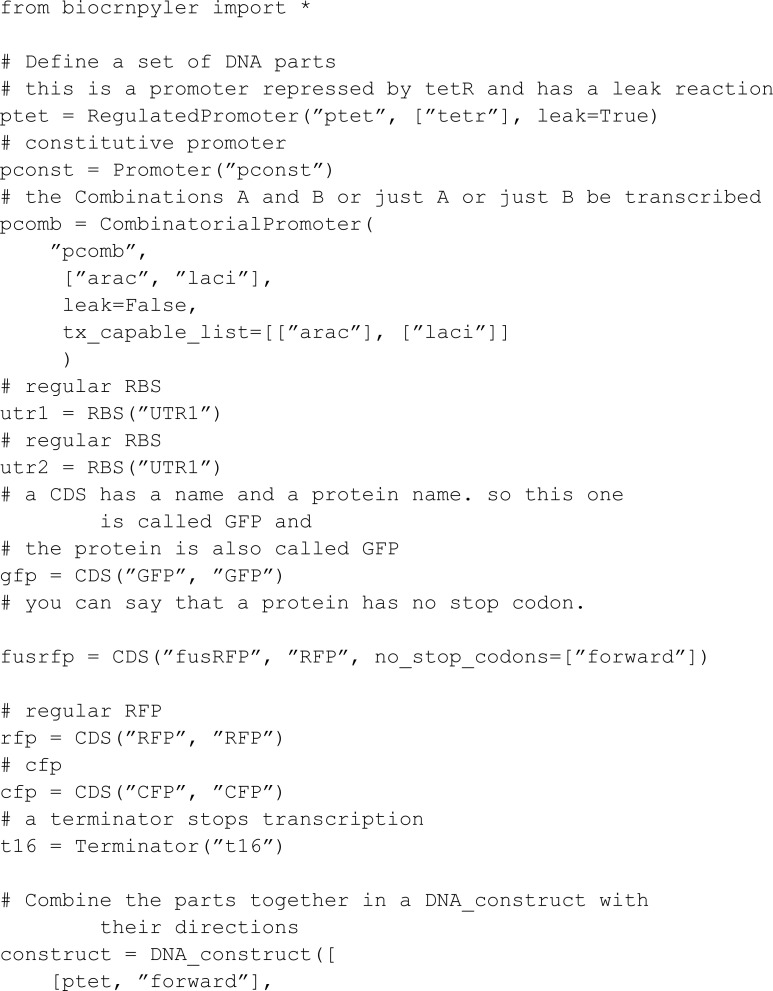





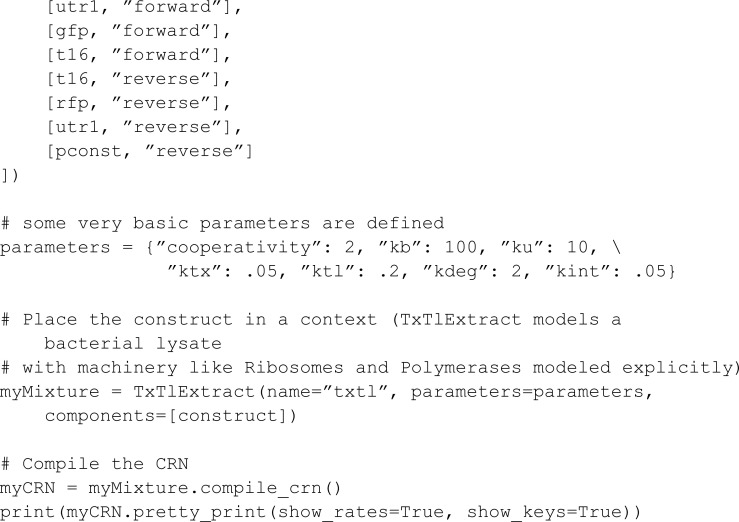



For this model, BioCRNpyler uses an automated reaction rate assignment strategy that relies on the names of the species. Such a strategy is not used in MobsPy. Thus, we set all rates arbitrarily and focus on syntax comparison. The model involves DNA translation and RNA transcription. For DNA translation, the DNA polymerase (DNA_Poly) binds to promoters (P2, Ptet) in a DNA strand and moves along the DNA to produce strands of messenger RNA (Mrna_P2, Mrna_Ptet). For RNA transcription, the ribosome (Ribo) binds to a messenger RNA and moves along it to produce proteins (GFP, RFP, CFP, GFP_F_RFP).

We start by defining the species:

from mobspy import *

Promoter, Start_Positions, Tet, Mortal = BaseSpecies()

Promoter.inactive, Promoter.active

Ribo, DNA_Poly = New(Start_Positions)

P2, Ptet = New(Promoter)

Mrna_P2, Mrna_Ptet, GFP, RFP, CFP, GFP_F_RFP = New(Mortal)

Further, we add death reactions:

# Death reactions

Mortal >> Zero [1]

In this model, promoters can either always be available for binding with the DNA polymerase (P2) or require binding with a BCRN species beforehand to bind to the DNA polymerase (Ptet). This is abstracted through the active and inactive characteristics:

# Promoter activation - only Ptet is activated. P2 is always

active Rev[Ptet.inactive + Tet >> Ptet.active][1, 1]

Translation and transcription are both implemented within a single function named read. DNA polymerase or ribosome meta-species are passed as the second argument of the function R for translation and transcription, respectively. The first parameter (Pro) is the binding target for R, being either a promoter located in the DNA strand for translation or a messenger RNA for transcription.

The product depends on the position where R initially binds on the DNA or RNA strand and where it stops reading. For instance, the protein GFP_F_RFP is generated when the ribosome begins at the GFP encoding position and continues into the RFP encoding without terminating, resulting in a fusion protein combining GFP and RFP. This is implemented inside the function using a for loop by passing the positions and respective products as the third argument strand.



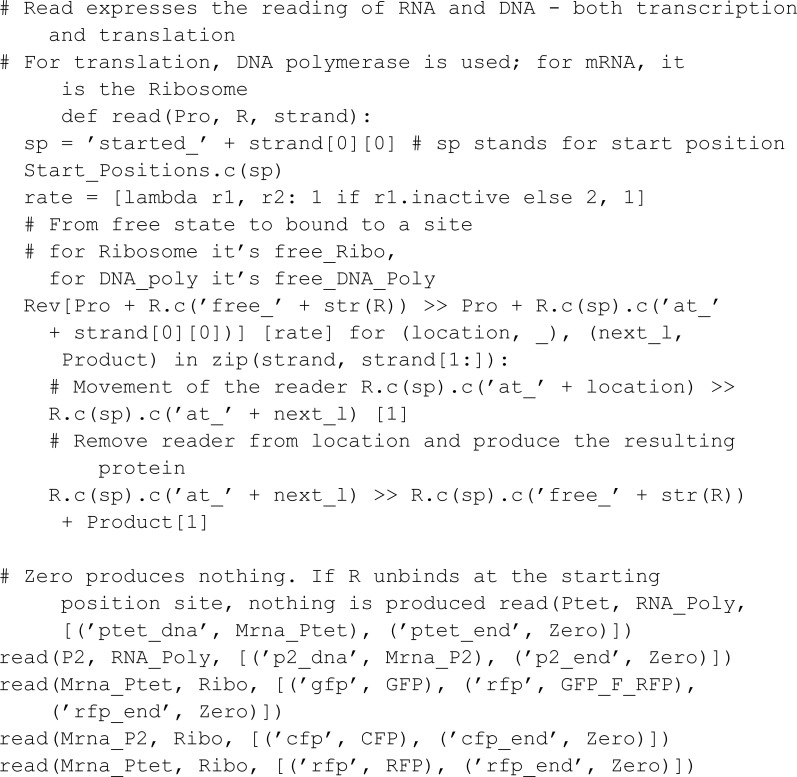



Finally, we assign initial values to meta-species.



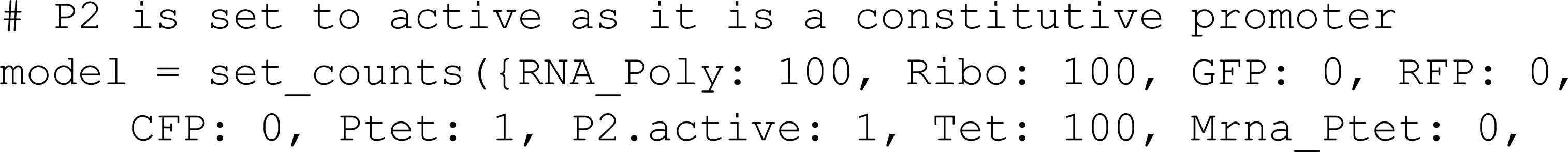





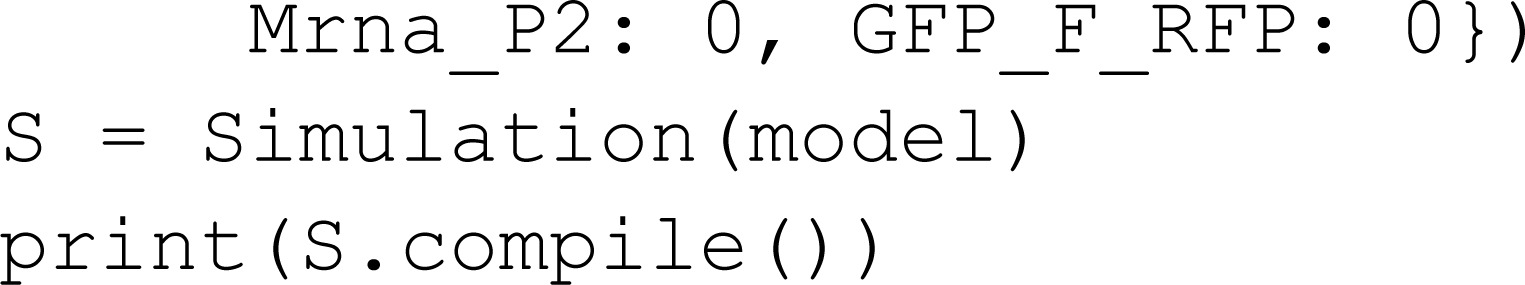



The resulting model has a similar length as the BioCRNpyler model. For MobsPy all the reactions are explicit in the model.

#### Comparison to PySB.

To compare MobsPy syntax to PySB syntax, we take the hello_pysb.py model from the PySB GitHub repository [36]:



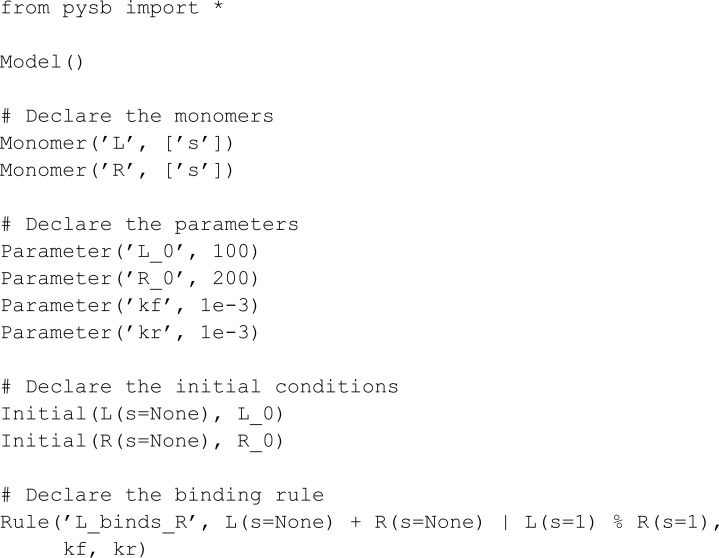



Implementing this model in MobsPy, we get:

from mobspy import *

L, R = BaseSpecies()

L_0, R_0, kf, kr = ModelParameters(100, 200, 1e-3, 1e-3)

L.sl_0 + R.sr_0 >> L.sl_1 + R.sr_1 [kf, lambda r: kr*r]

L(L_0), R(R_0)

S = Simulation(L | R)

print(S.compile())

Multiple differences in syntax choices are evident in this comparison. Firstly, the BaseSpecies constructor in MobsPy allows for the simultaneous definition of multiple meta-species, streamlining the process of declaring several monomers simultaneously. Additionally, characteristics that define bindings are implicitly added to reactions, eliminating the need for users to manually define these characteristics when creating base species and typing them again in subsequent reactions.

PySB automatically generates Python variables using the string names provided in its Monomer constructor, blending string literals with variable references. While this approach works and compiles correctly, it can lead to namespace pollution. Namespace pollution occurs when dynamically injected variables create conflicts or unintended overwrites, especially in environments where multiple modules are imported or interact. It can result in hard-to-debug issues, as variable name conflicts may propagate across modules [37].

MobsPy achieves the same functionality by using the variable name itself as the internal string representation. This eliminates the need for the user to manage or interact with string representations directly. Instead, users work exclusively with variables. Also, users can import MobsPy’s global variables with import mobspy as ms instead of from mobspy import * if namespace pollution is ever presented as an issue.

On the other hand, PySB has more synthesis capabilities due to its high-level modularity features. For MobsPy to achieve a similar level of syntax reduction, a necessary step is to implement ways to combine high-level models.

#### Comparison to BioNetGen.

In this section, we compare MobsPy to BioNetGen. To differentiate with the comparison with Kappa, we chose a multi-state model available in VCell’s introductory example list [24, 38]:



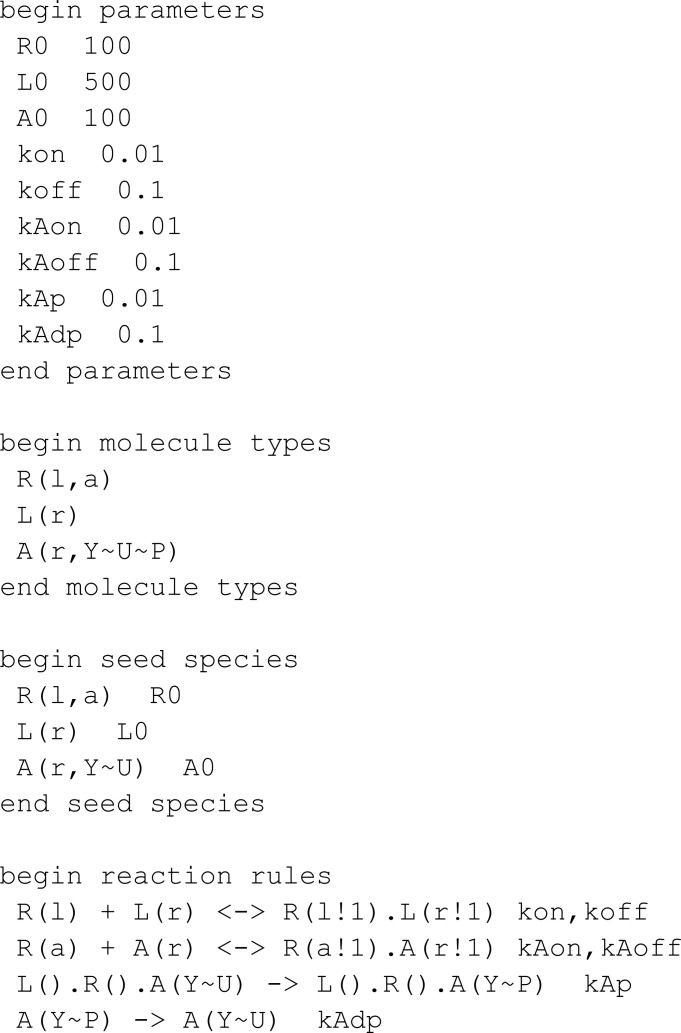





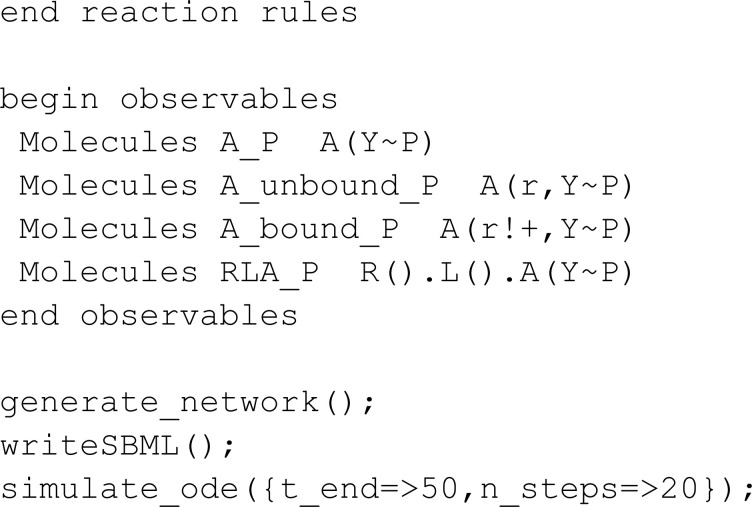



Translated into MobsPy, this model becomes:



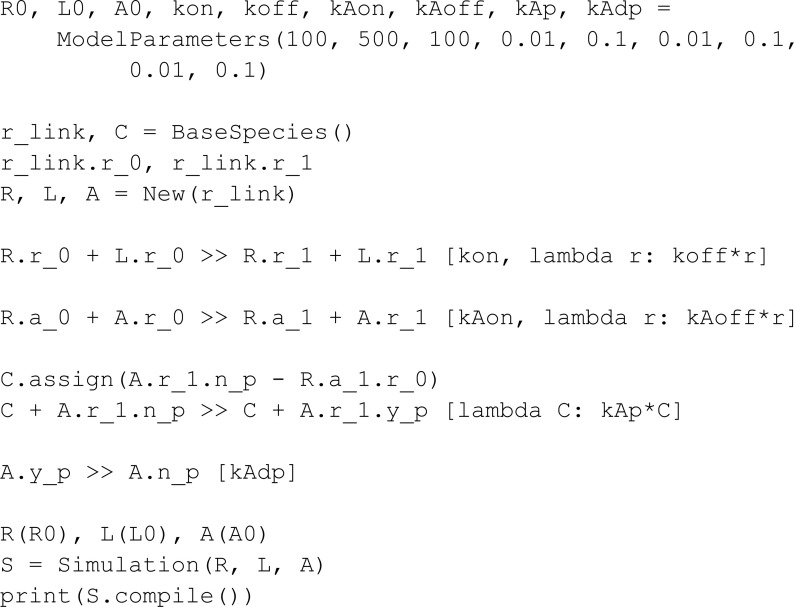



Some syntax advantages gained from inheritance can be seen in the definition of R, L, A. Since all three inherit from r_link, there is no need to repeatedly redefine binding states when creating the molecules. Furthermore, MobsPy implicit state definition is used to define:

- Phosphorylation of A is represented by y_p (phosphorylated) and n_p (unphosphorylated).
- R binding to A is a_0 (unbound) and a_1 (bound).

Finally, observables do not need to be explicitly defined in MobsPy. Instead, the user can query the simulation results directly from the generated data.

In the third meta-reaction, which is complex-based, BioNetGen uses its graph rewriting algorithm to identify and count complexes, defining the reaction rate accordingly. In MobsPy, we achieve the same effect by explicitly defining a complex species (C) assigned the number of receptor-ligand complexes containing unphosphorylated A. The reaction rate is based on C, ensuring that only A.n_p inside a complex contributes to the phosphorylation reaction, rather than all A.n_p in the system. This expression is simple to write for small complexes. However, as the complex grows in size, so does the expression in the assignment.

It is vital to note that this model considers the binding rate of molecules linked to other parts of the complex equal to the binding rate of the molecules when they are not bound. One example lies in the dephosphorylation of A, which is of equal rate when A is in a complex or not. It qualifies as an approximation, as in most chemical systems, the binding of other complexes and the size of the complexes affect the reaction rate constant both due to electromagnetic interactions between adjacent bond sites and changes in overall complex mass [39–41].

#### Comparison to antimony.

Unlike the other languages presented in this section, Antimony does not provide reaction modularity. Instead, it serves as a human-readable alternative to SBML, offering additional features such as modifying the CRN structure mid-simulation[18]. Since MobsPy supports Antimony model generation through the generate_antimony method, we employ this feature for comparison.

The following MobsPy script generates an Antimony model:

from mobspy import *

Replicator, Mortal = BaseSpecies()

Replicator >> 2*Replicator [lambda r: 2*(100 - r)*r]

Mortal >> Zero [1]

A, B, C = New(Replicator*Mortal)

A + B >> C [1]

S = Simulation(A | B | C)

print(S.generate_antimony()[0])

This script produces the following Antimony model:

model mobspy_25681

A = 0 dimensionless

B = 0 dimensionless

C = 0 dimensionless

_vol = 1 dimensionless

reaction_0: A -> ; A * 1

reaction_1: B -> ; B * 1

reaction_2: C -> ; C * 1

reaction_3: A + B -> C; A * B * 1 * _vol^-1

reaction_4: A -> 2 A; ((2*(100-A))*A)

reaction_5: B -> 2 B; ((2*(100-B))*B)

reaction_6: C -> 2 C; ((2*(100-C))*C)

end

This output demonstrates how MobsPy expressions integrate with Antimony, as both directly encode reaction rates using mathematical expressions.

## Availability and future directions

MobsPy is freely available at the Python Package Index (via pip) and its GitHub repository:

https://github.com/ROBACON/mobspy

Future updates will focus on enhancing modularity by developing a library of reusable meta-species models, particularly for translation and transcription. Additionally, we aim to refine the syntax to improve integration with existing models. Finally, we plan to enhance the current geometry models by introducing methods that simplify mesh definition while mitigating state space explosion. Finally, we aim to implement a syntax that automates complex-based assignments.

## Supporting information

S1 Appendix Implementation details and plot configurations (PDF)
