# Supplementary material for: MobsPy: A programming language for biochemical reaction networks
Source: PLoS Comput Biol. 2025 May 19;21(5):e1013024. doi: 10.1371/journal.pcbi.1013024 (PMC12165391; doi:10.1371/journal.pcbi.1013024)
Supplement: S1 Appendix — (PDF) [file pcbi.1013024.s001.pdf]

Appendix

Implementation Details

A meta-species is represented by a `Species` object. Among others, the object contains the set of its predecessor species from which it was constructed via multiplication, the set of characteristics that were added to it, and the set of meta-reactions (as `Reaction` objects) this meta-species is involved in. When a characteristic is accessed via the dot notation, the object checks the states of all its predecessor meta-species. If the characteristics are absent, they are added.

`Species` that appear in a reaction generate `ReactingSpecies` objects. At their core is a list of dictionaries, storing their meta-species, characteristics, and stoichiometry in the reaction.

Before the simulation, MobsPy compilation is internally triggered, collecting all reactions and species. Per reaction, the compiler loops through all `ReactingSpecies`, collecting their predecessor meta-species and generating its set of species, taking into account dot operators to refine the set. For optimization of compile time, MobsPy internally uses two dictionaries: from characteristics to meta-species and a reverse lookup. The output of the compilation process is an SBML string of the model. If a user triggers the execution of the `run` method from the `Simulation` object, MobsPy simulates the generated SBML model using BasiCO. Direct access to the SBML model is possible via the `generate_sbml` method and allows for simulation with other backends.

After simulation with an SBML backend, simulation results are mapped to meta-species and plotted either via a simplified hierarchical plotting library or using standard libraries, e.g., Pandas. The default MobsPy stochastic plot parametrization generates two figures for each species, plotting the concentrations and their mean and standard deviation over time.

Internal plotting parametrization is in three layers: Parameters in the first level, for example, `linewidth`, are set for all figures. The second level (`figures`) contains a list of figures. Parameters given in this list will be set in the respective figure. The lowest level (`plots`) allows one to set parameters for individual curves inside a figure. Lower levels take precedence over higher levels. Additionally, species-specific parameters, such as the color and style used for a species plot, can be set by using the species name as a key. These species keys can be used at any level and will follow the hierarchical rules of the parameters.

Plot Configurations

Plots are configured via Python dictionaries. An example plot configuration for the Donor and Phage Receiver model is shown below.

```
{
  "R1":{"color": "b", "label": "R1"},
  "R2":{"color": "g", "label": "R2"},
  "Receiver.not_infected": {"color": "r", "label": "Receiver.not_infected"},
  "Receiver.early_infection": {"color": "k", "label": "Receiver.early_infection"},
  "Receiver.late_infection": {"color": "c", "label": "Receiver.late_infection"},
  "Donor":{"color": "b", "label": "Donors"},
  "Antibiotics": {"color": "m", "label": "Antibiotics"},

  "tight_layout": true,
  "title_fontsize": 14,
  "pad": 1,

  "xlabel": "Time (hours)",
  "ylabel": "Conc. (mL\(^{-1}\))",
  "xlabel_fontsize": 12,
  "ylabel_fontsize": 12,
  "vertical_lines":[0.56],
  "time_filter": [0, 1.5],

  "figures": [{
    "title": "Biphase Resource Availability",
    "species_to_plot": ["R1", "R2"],
     "annotations": [{"text": "S1", "coordinates": [0.48, 1.5e11], "fontsize": "large"},
             {"text": "S2", "coordinates": [0.58, 1.5e11], "fontsize": "large"}]
  },{
    "title": "Resulting Infection",
    "species_to_plot": ["Receiver.not_infected", "Receiver.late_infection",
      "Receiver.early_infection", "Antibiotics"],
    "annotations": [{"text": "S1", "coordinates": [0.48, 3e9], "fontsize": "large"},
            {"text": "S2", "coordinates": [0.58, 3e9], "fontsize": "large"}]
  }]
}
```
