# Supplementary material for: MobsPy: A programming language for biochemical reaction networks
Source: PLoS Comput Biol. 2025 May 19;21(5):e1013024. doi: 10.1371/journal.pcbi.1013024 (PMC12165391; doi:10.1371/journal.pcbi.1013024)
Supplement: Attachment — Submitted filename: Reviewer response.pdf [file pcbi.1013024.s002.pdf]

Dear Reviewers,

We thank you for the many insightful comments. We respond to the individual comments below.

Reviewer #1

The article presents MobsPy, a Python-based language designed to simplify the modeling of Biochemical Reaction Networks (BCRNs). The authors introduce the concept of meta-species and meta-reactions to manage the complexity of BCRNs, enabling arguably more concise and manageable models. The paper highlights the challenges of modeling complex biochemical systems and proposes MobsPy as a tool to reduce errors and simplify the creation and maintenance of models. While I agree that the creation of models using species characteristics will be much easier using MobsPy, I'm not sure that parameterization, annotation and maintenance will also be simplified.

We agree with the reviewer that our goal was on simplifying the creation of models using species, reactions, and characteristics as well as simulation of such models without having to manually manage mappings of species to meta-species. We are not sure to understand if the reviewer refers to automated parametrization or the ease of setting parameters in the MobsPy.

Concerning the first, automated parametrization of models was added as a feature to primarily access BasiCO parametrization procedures at this stage. We plan to extend this in a future version, however. We are also using automated parametrization via scipy optimizing the loss of a MobsPy model w.r.t. measured data. This is not integrated into the package yet, however.

Considering the parametrization, we have implemented a function called ModelParameters which is capable of defining parameters for rates and species concentrations/counts in the model. However, by default, we do not define rate constants as parameters to both simplify the resulting SBML file and to facilitate the introduction of non mass-action kinetics rates which use multiple parameters simultaneously.

The article provides a thorough comparison with other frameworks, effectively highlighting MobsPy's advantages. Though I found it cumbersome to have to look up the original examples from their respective websites. One aspect I miss in the translated MobsPy version are the actual parameter names used to make it comparable (so for example:
A >> 2*B [mobspy_parameters.Mobspy_Parameter('k1', 3)]
B >> C + D [mobspy_parameters.Mobspy_Parameter('k2', 1.4)]
)

The code above is not the intended syntax used for MobsPy parameters (the XOR Gate model in the publication). MobsPy creates SBML translatable parameters in a similar way to meta-species, using the variable name as a parameter name with the possibility of renaming them to the user's liking when necessary.

We intended the used to write the same example above as:

```
k1, k2 = ModelParameters(3, 1.4)

A >> 2*B [k1]
B >> C + D [k2]
```

which will name the parameters k1 and k2 in the SBML: After testing your initial model and visualizing the resulting translated SBML file, we see that the parameters names matched your provided string.

. It would have been nice if the authors had chosen a direct comparison with PySB as well.

We thank the reviewer for the suggestion and we added a comparison to PySB. As MobsPy focuses on syntax simplification, we followed a similar approach to the other compared tools and used a simple PySB model and implemented it in MobsPy. We discuss our conclusions and point out the differences.

The software itself is easily installable and the github repository contains the examples and works as advertised.

The main questions left for me are:

• The MobsPy language uses '>>' to denote an irreversible transition. Is there a way to define a reversible one?

Yes, reversible reactions are expressed with the Rev[reaction][forward rate, backward rate] operator. We have added a sentence in Section 3.1 and added a paragraph and an example to the GitHub README shown on the main GitHub page of MobsPy. Furthermore, inspired by your comment, we added a new syntax for reversible reactions, where the user can define a reversible reaction by simply supplying two rates [forward rate, backward rate] and a notebook describing reversible reactions.

• It would be nice if in the compiled model, there would be a way to easily use parameters instead of the number literals. This becomes especially important later for parameter estimation or entering values from literature for specific reactions. Is there a shortcut for that?

We appreciate the reviewer's request and believe it is indeed an extremely useful feature. Therefore, we added a function called update_model that can both update parameter values and specific initial concentrations using MobsPy's query syntax. We have also added a tutorial describing how to use this function. We also added new test functions to our test script for this feature.

• It would be easier to use the software if there was a detailed readthedocs site with the examples and api description. I see the authors started with one, it would be great to see it succeed.

Thank you for this suggestion. We finished the readthedocs site for mobspy and refer to it in the GitHub README. The page is available at https://mobspy-doc.readthedocs.io/en/latest/

• It would also be nice if the examples / notebooks covered the SBML export explicitly. Especially together with using non-default rate laws.

We thank the reviewer for their comment and added a notebook describing how to export models to SBML and Antimony. Further, we added an example with non-mass-action kinetic rates.

• Looking at the SBML generated, I see a need for improvement:
o The exported sbml contains a dimensionless volume parameter, that is used instead of SBML compartments (though the exported compartment 'c1' is also dimensionless). This will make

multi-compartment modelling difficult and error prone later, when the authors work on model composition.
o In fact, the whole SBML generated is unitless since dimensionless units are also used for species. It would be nice if the units (which the authors recognize as important since they added support for them for the mobspy language through pint) could also be exported to the SBML.

We thank the reviewer for their comment, to show that compartments are already implemented through meta-species and the MobsPy syntax, we changed the following lines in the XOR gate model from the paper:

```
with Compartment.c1 :

        A >> B + C [1]
```

Which translates to:

```
        A.c1 >> B.c1  + C.c1 [1]
```

Furthermore, model composition strategy is already implemented through the sum of simulation objects. We also plan to work on model composition using MobsPy meta-species objects, instead of the SBML model files.

We agree that currently MobsPy:

- Does not explicitly use compartments in the generated SBML model. Instead, compartments are reserved as inheritors of meta-species rather than being mapped directly to SBML compartments.
- Unit handling is done at the Python-level not at the SBML level. All units are converted to a documented base system to facilitate checking for the consistency of expressions that use multiple parameters and constants with units. Further it allows for a simpler integration of models who use both counts and concentrations simultaneously. The decision to keep volume dimensionless (set to 1) ensures that counts and concentrations remain equivalent under this condition.

• MobsPy also exports Antimony script, however, the generated script is unfortunately also invalid. The reaction scheme generated is invalid. For example reaction schemes like 'A B -> 2*C D;..' are created, when it should be 'A + B -> 2 C + D;…'

We appreciate the reviewer bringing this to our attention. This feature was introduced after the initial submission, and we corrected the issue before accessing this review. We have now ensured that the latest version works as intended.

• Consistent spelling should be used for the main concepts like meta-species and it should be consistently hyphenated.

Thanks. We checked main concepts like meta-species, meta-reactions, and MobsPy in the paper for consistent writing and hyphenation. We also reworked the writing in the git repo (README and tutorial examples) for consistency with the paper.

• For roadrunner and basico the publications for the tools should be used rather than their website.

We agree. Both references were updated to the publications.

Reviewer #2

Manuscript describes an agent-based approach for modeling, where the authors introduced multi-state agents that are termed by authors as “meta-species”, and interactions between meta-species that generate the reaction network to be simulated stochastically.

The approach is very interesting and should be useful for many systems biology modelers. However, the present manuscript suffers from a major drawback – the authors don’t compare their approach to the existing approaches and tools that aim at the same target - designing a language to simplify the modeling processes. Because of that, the manuscript has multiple overstatements, missing citations, or misrepresents other software tools.

We thank the reviewer for their comment. We went over every such statement and removed several statements about other languages that we believe to be inaccurate. For instance, the statement about MobsPy being able to copy Kappa’s syntax capabilities. We have corrected it based on your comments and focused on expressing the differences.

1. Regarding human-readable description, the Antimony serves the same purpose. A significant part of the manuscript talks about assignments, events, and other elements of SBML modeling. It would be useful to compare with Antimony (providing the same model coded in both languages) and state the advantages of Antimony (e.g. better coverage of SBML) and mobspy (elements of rule-based modeling).

We thank the reviewer for their comment. We added a comparison section for Antimony as requested.

2. Regarding the introduction of meta-species, it’s a reinvented name of “multi-state molecule” that was introduced first by STOCHSIM back in 2001 (doi:10.1093/bioinformatics/17.6.575). The first general-purpose software that used multi-state molecules was BioNetGen software (doi:10.1093/bioinformatics/bth378), which seems to be roughly equivalent to the MobsPy declaration of meta-species. The BioNetGen in 2004 expanded the rules of interactions between multi-state species into the reaction network. The tricks with link1 and link2 were possible, but any complexes of several connected molecules were difficult to model, leading to the need to introduce bonds in addition to states, as described in doi: 10.1007/978-1-59745-525-1_5.

We kindly thank the reviewer for the comment. Multiple comparisons were introduced to the paper with multi-state molecules to better express their differences. Furthermore, we tried to clarify what makes MobsPy unique in the statement that follows:

- MobsPy meta-species are called meta-species because much like meta-classes in python they require the combination and operation between objects to create new classes. MobsPy not only provides this capability of combining species to generate new species, allowing for both the introduction of reaction inheritance and re-utilization of states throughout multiple species, but also forces the user to combine species to create higher dimensional species. For instance, the line in your provided model that defines a tree:

  Tree(age~young~old,color~green~yellow~brown,location~dense~sparse)

is impossible in MobsPy as it requires the combination of three meta-species. Further, the states and reactions of the species used to define three, can be used by other species through inheritance.

Furthermore, such definition streamlines the definition of observables, as meta-species can just be queried on the results object to find whatever the user needs.

We believe this key difference is enough to distinguish both notions.

3. The authors should compare rule-based features of MobsPy to those of BioNetGen and Kappa. Modeling of complex formation with MobsPy is much trickier. Including several constraints into the interactions (A can bind to C if A is bound to B and C has a phosphorylation state and is in a complex with other molecules) is tricky if possible. Modeling of polymers is impossible. However, MobsPy excels in modeling just multi-state molecules, without binding. For example, the reaction A.state1 -> A.state1 + A is easy in MobsPy. However, both BioNetGen and Kappa can only match one reactant to one product, so the second A will give an error if not defined, which requires multiple reaction rules (r3-r3_4) to define all combinations. See below the BioNetGen model that implements the tree model:

```
begin model
begin molecule types
Tree(age~young~old,color~green~yellow~brown,location~dense~sparse)
Dead_tree()
end molecule types
begin seed species
Tree(age~young,color~green,location~dense) 25.0
Tree(age~old,color~green,location~dense) 25.0
Tree(age~young,color~green,location~sparse) 25.0
Dead_tree() 0.0
Tree(age~old,color~green,location~sparse) 25.0
end seed species
begin observables
Molecules All_Trees Tree()
Molecules Young_trees Tree(age~young)
Molecules Brown_trees Tree(color~brown)
Molecules Dense_trees Tree(location~dense)
Molecules Sparse_trees Tree(location~sparse)
end observables
begin reaction rules
r00: Tree(age~young) -> Tree(age~old) 0.1
r01: Tree(age~old) -> Dead_tree() 0.1
r02: Tree(age~old,location~dense)%1 + Tree(age~young,location~dense) ->
Tree(age~old,location~dense)%1 1.0E-10
r03: Tree(age~old,color~green,location~dense)%1 -> Tree(age~old,color~green,location~dense)%1 +
Tree(age~young,color~green,location~dense) 0.1
r03a: Tree(age~old,color~green,location~sparse)%1 ->
Tree(age~old,color~green,location~sparse)%1 + Tree(age~young,color~green,location~sparse) 0.1
r03b: Tree(age~old,color~yellow,location~dense)%1 -> Tree(age~old,color~yellow,location~dense)%1
+ Tree(age~young,color~yellow,location~dense) 0.1
r03c: Tree(age~old,color~yellow,location~sparse)%1 ->
Tree(age~old,color~yellow,location~sparse)%1 + Tree(age~young,color~yellow,location~sparse) 0.1
r03d: Tree(age~old,color~brown,location~dense)%1 -> Tree(age~old,color~brown,location~dense)%1
+ Tree(age~young,color~brown,location~dense) 0.1
```

```
r03e: Tree(age~old,color~brown,location~sparse)%1 ->
Tree(age~old,color~brown,location~sparse)%1 + Tree(age~young,color~brown,location~sparse) 0.1
r04: Tree(color~green) -> Tree(color~yellow) 10.0
r05: Tree(color~yellow) -> Tree(color~brown) 10.0
r06: Tree(color~brown) -> Tree(color~green) 10.0
end reaction rules
end model
```

We thank the reviewer for the time and help by providing us the translated model. Following the reviewer's suggestion, we have added an additional reaction to the Tree model to further illustrate MobsPy's distinctions from Kappa and BioNetGen.

4. Implementation and use of MobsPy should be compared to PySB – whether the same model can be modeled in PySB and MobsPy, and which is easier to use.

We thank the reviewer for the comment and suggestion. Similarly to Kappa and BioCRNpyler, we added a PySB introductory model and compared the syntax of the two languages.

5. I recommend comparing MobsPy to BIOCHAM (http://contraintes.inria.fr/biocham) which works with multi-state molecules. I also recommend looking at ML-rules (doi: 10.1007/978-1-4939-9102-0_6)

We thank the reviewer for the suggestion. To address this, we added a paragraph in Section 4.2 that compares MobsPy with other multi-state languages focusing on the benefits of inheritance.

6. Some statements in the manuscript are incorrect:
a. Page3: "Common to the above tools, however, is that they lack means for abstraction at the reaction level". What do authors mean by "abstraction"? All these tools provide visualization of the reaction network, much better than MobsPy code. If rule-based features are counted as "abstraction", then there are other tools that have them, e.g. SmolDyn (doi: 10.1093/bioinformatics/btw700) and VCell (doi:10.1093/bioinformatics/btw353) that implement BioNetGen muti-state rule-based modeling. CellDesigner implements some multi-state features.

We apologize for any confusion caused by our use of the term "abstraction." In our manuscript, we align with the terminology used by BioCRNPyler, where "abstraction" refers to the concept of reaction modularity. Specifically, we define abstraction as the strategy employed to streamline the scripting of reactions, enhancing modularity and reusability. Based on your comment, we have changed the sentence to better explain ourselves.

b. Page 3: "Unlike rule-based languages, MobsPy's meta-species characteristics can be virtually anything assigned by the user, accommodating a wide range of possibilities according to intent" – the same is true for rule-based approach, the term "molecule" should not confuse – it's just an object that can be anything.

We agree with the reviewer and removed the sentence.

c. Page4: Parameter sweeps, utilization of multiple CPUs are the features of Python and BasiCO, not MobsPy. PySB can do the same.

We appreciate the reviewer's feedback and have clarified in the manuscript that parametric sweeps and multi-threading are features of the MobsPy Python module rather than MobsPy itself. MobsPy provides a reserved syntax to define parametric sweeps efficiently, similar to BioNetGen, by assigning multiple values to a parameter.

d. Page 15, description of Kappa, BioNetGen, and pySB: "the characteristics of a species, or, equivalently, its state, are limited to the occupancy status of their binding sites" – wrong, there are states that are not limited to occupancy, see the BioNetGen example I demonstrated above.

We agree with the reviewer and thank them. We removed the statement from the manuscript

e. Page 16: "PySB, although lacking built-in simulation capabilities" – the same is true for MobsPy which lacks simulation capabilities and uses external simulators.

We thank the reviewer for their comment. We removed the statement from the manuscript.

f. Page 16: "Current tools that provide reaction modularity, like Kappa and BioCRNPyler [27, 10], do not use built-in units" – wrong, rule-based modeling with VCell uses built-in units. The Tree model in the manuscript provides a plot in counts while GitHub example results in simulation results in concentrations – ironically as the authors talk about how great their tool is with units.

We appreciate the reviewer's feedback. We changed the statement to make it clear that with the addition of VCell both Kappa and BioNetGen rely on the modeler to ensure unit consistency. MobsPy provides unit consistency by converting rates, concentrations, and counts to a standardized system and validating rate expression units. We changed concentrations to counts in the tree GitHub example.

7. The models described in the manuscript don't match the simulation plots, and do not match the tutorials at GitHub. I started to deal with the first "tree" example and I tried to reproduce it in BioNetGen just from the manuscript. It was impossible: initial conditions start from 75, while plots show 100. My simulations have at 20 years total trees down to 20%, while they didn't change in the manuscript plots. Then I loaded tutorial number 1, and got simulation results matching my BioNetGen simulations but not in the manuscript. Next, I loaded a model from Examples folder – and got simulation results matching "qualitatively" plots in the manuscript but not numbers in the code snippets in the manuscript. I had to go line by line to figure out the difference in numbers (the death rate is 10 times higher in the tutorial than in the manuscript text and the first tutorial notebook).

We appreciate the reviewer's comments and apologize for this. We decided to add the journal models to the MobsPy GitHub to facilitate your verification. They are under docs/example_models/journal_models.

8. Same with all other examples. The reference [22] does not have the plots given in this manuscript – how are readers supposed to believe that the simulations are correct?

We appreciate the reviewer's concern. We have clarified in the text that this work builds upon our own prior research.

As conclusion, I would appreciate:
- Detailed comparison of MobsPy to other tools that have multi-state features, listing pros and cons. Some of the tools that use rule-based technology are listed at https://bionetgen.org/software

We thank the reviewer for the suggestion. Several comparisons with multi-state features have been introduced and updated.

- Choose several tools (most obvious PySB, BioNetGen and/or Kappa) and demonstrate models/features that can be simulated by all tools (including simulations and plots), and features that are unique to MobsPy. You can use https://bionetgen.org/applications and https://bnglviz.github.io/examples.html as a collection of examples that are covered by BioNetGen modeling.

We appreciate the reviewer's suggestion. We added more comparisons.

- All code snippets in the manuscript should match precisely those in the examples folder

- All plots in the manuscript should be precisely reproducible from the examples in the GitHub.

- Names of models in the examples folder should be given in the manuscript. Now it's a quest to find the matching model.

- If a model claims to reproduce the published results, please point out to the exact figure in the original manuscript you reproduce.

We thank the reviewer for their comment. To facilitate verification and ensure easier access:

- We have added the journal models to the MobsPy GitHub repository, ensuring that all models referenced in the manuscript are easily accessible and verifiable.
- The file names in the repository have been updated to correspond exactly to their matching models in the manuscript, making it easier for readers to locate and compare them.
- All code snippets in the manuscript have been checked for consistency with the examples provided in the repository.
- The plots in the manuscript can now be precisely reproduced from the corresponding example models in GitHub.

Minor issues:
1. Colors are completely decoupled from the rest of the Tree model – every modeler would just create a separate model for color transition. A simple model where colors will depend on other features like age will be much more interesting.

We appreciate the reviewer's feedback. The Tree model is intended as a toy example to illustrate MobsPy's syntax and structuring capabilities rather than to model a biologically or physically detailed system. While more complex dependencies, such as color transitions based on age, could certainly be implemented, the current example was chosen for its clarity and didactic value.

2. I got strange warning messages when running notebook 1:
Compiling model

WARNING: The following reaction:
{'re': [(1, 'Tree_dot_brown_dot_dense_dot_old')], 'pr': [(1, 'Tree_dot_brown_dot_dense_dot_old'), (1, 'Tree_dot_brown_dot_dense_dot_young')], 'kin': 'Tree_dot_brown_dot_dense_dot_old * 3.168808781402895e-09'}
Is doubled. Was that intentional?

We thank the reviewer for the comment. This means an equal reaction was added twice to the same meta-species, as this can be the result of running the model in separate cells in Jupyter Notebook, we have decided to provide a warning.

3. Please delete numbers in the code snippets in the manuscript – it makes copying to Python complicated.

We removed line numbers from the code.
